# Salmonid polysialyltransferases to generate a variety of sialic acid polymers

**DOI:** 10.1038/s41598-023-42095-0

**Published:** 2023-09-20

**Authors:** Mathieu Decloquement, Marzia Tindara Venuto, Virginie Cogez, Anna Steinmetz, Céline Schulz, Cédric Lion, Maxence Noel, Vincent Rigolot, Roxana Elin Teppa, Christophe Biot, Alexander Rebl, Sebastian Peter Galuska, Anne Harduin-Lepers

**Affiliations:** 1 Univ. Lille, CNRS, UMR 8576 - UGSF - Unité de Glycobiologie Structurale et Fonctionnelle, 59000 Lille, France; 2 Institute of Reproductive Biology, Research Institute for Farm Animal Biology (FBN), Wilhelm-Stahl-Allee 2, 18196 Dummerstorf, Germany; 3 Institute of Genome Biology, Research Institute for Farm Animal Biology FBN, Wilhelm-Stahl-Allee 2, 18196 Dummerstorf, Germany; 4 Unité de Glycobiologie Structurale et Fonctionnelle, UMR CNRS 8576, Faculté des sciences et Technologies, Univ. Lille, 59655 Villeneuve d’Ascq, France

**Keywords:** Biochemistry, Biological techniques, Molecular biology

## Abstract

The human polysialyltransferases ST8Sia II and ST8Sia IV catalyze the transfer of several Neu5Ac residues onto glycoproteins forming homopolymers with essential roles during different physiological processes. In salmonids, heterogeneous set of sialic acids polymers have been described in ovary and on eggs cell surface and three genes *st8sia4, st8sia2-r1* and *st8sia2-r2* were identified that could be implicated in these heteropolymers. The three polysialyltransferases from the salmonid *Coregonus maraena* were cloned, recombinantly expressed in HEK293 cells and the ST8Sia IV was biochemically characterized. The MicroPlate Sialyltransferase Assay and the non-natural donor substrate CMP-SiaNAl were used to demonstrate enzyme activity and optimize polysialylation reactions. Polysialylation was also carried out with natural donor substrates CMP-Neu5Ac, CMP-Neu5Gc and CMP-Kdn in cell-free and cell-based assays and structural analyses of polysialylated products using the anti-polySia monoclonal antibody 735 and endoneuraminidase N and HPLC approaches. Our data highlighted distinct specificities of human and salmonid polysialyltransferases with notable differences in donor substrates use and the capacity of fish enzymes to generate heteropolymers. This study further suggested an evolution of the biological functions of polySia. *C. maraena* ST8Sia IV of particular interest to modify glycoproteins with a variety of polySia chains.

## Introduction

The nine-carbon α-keto acidic monosaccharides *N*-acetylneuraminic acid (Neu5Ac), *N*-glycolylneuraminic acid (Neu5Gc) and 2-keto-3-deoxy-nonulosonic acid (Kdn) are the three most common sialic acids (Sias) found in vertebrates, except in human tissues predominantly featuring Neu5Ac residues^1^. Sias at the outmost end of glycolipids and glycoproteins are linked to galactose (Gal) and *N*-acetylgalactosamine (GalNAc) via α-2,3 or α-2,6 bonds or linked to α-2,8 on other Sias forming anionic linear polymers named α-2,8-polysialic acids (polySia). The structural diversity and complexity of polySia is based on their degree of polymerization (DP), the nature of Sias and their possible modifications^2^. Homopolymers of Neu5Ac residues found on the neural cell adhesion molecule (NCAM) has been intensively studied in the mammalian central nervous system where it plays an essential role during post-natal brain development^3,4^. However, polysialylation takes place also in other organs^5^. For instance, in the male reproductive system, polySia is involved in the postnatal differentiation process of peritubular smooth muscle cells^6,7^. Moreover, polySia was found in semen and may influence immunological processes in the female reproductive tract^8^.

In contrast to mammals, a broad polySia diversity has been described in salmonid fish. Seminal glycomics studies have shown the presence of homo- and hetero-polymers containing Neu5Ac and/or Neu5Gc or Kdn residues with a DP ≤ 25 Sia residues^9–11^ potentially capped by Kdn^12^. In cortical alveoli of salmonid eggs, the polysialylated glycoprotein (PSGP) seems to be the major carrier of polySia^9–11,13^ and these polySia chains have been implicated in sperm acrosomal reaction preventing polyspermy during fertilization^14,15^. Moreover, these polySias, which are α2,6-linked to the inner GalNAc residue of *O*-glycans, provide a protective effect against pathogens in the reproductive system^16^. More recent studies pointed to the presence of large quantities of polySia in fish serum^17^ and also on various fish oogonia^18^ despite different poly-α-2,8-sialyltransferases setups^19^.

**Figure 1:**
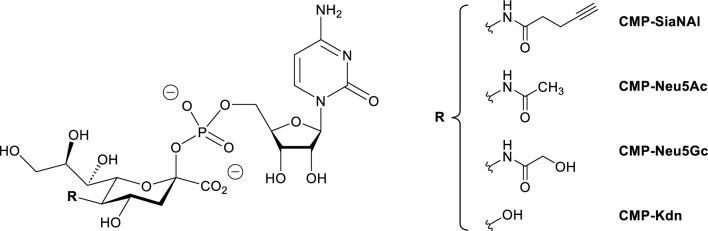
Chemical structure of the various CMP-Sias synthetized and used in this study: CMP-Neu5Ac, CMP-Neu5Gc, CMP-Kdn and CMP-SiaNAl.

Biosynthetic enzymes (*i.e.* sialyltransferases) catalyzing the transfer of Sia residues from an activated donor substrate CMP-sialic acid (CMP-Sia, Fig. 1) onto glycolipids or glycoproteins are classified into ST3GAL, ST6GAL, ST6GALNAC and ST8SIA^20,21^. Phylogenetic distribution of sialyltransferases was assessed in Metazoan and found to be extremely diverse in teleost fishes^19,22,23^. Two poly-α-2,8-sialyltransferase genes *ST8SIA2* and *ST8SIA4* of the ST8SIA family have been described in the human genome^22^, whereas homologous genes in fish genomes show an uneven distribution^19^. In salmonids like the whitefish *Coregonus maraena* (*C. maraena*) or *Oncorhynchus mykiss* (*O. mykiss*)*,* in addition to *st8sia4*, two *st8sia2*-related genes were identified as a result of the species-specific genome duplication event R4-SGD that took place 80 million years ago. These genes showed distinct expression profiles in fish tissues with *st8sia2r-1* and *st8sia2r-2* genes displaying their highest mRNA levels in gonads and telencephalon, and in gonads and spleen, respectively, and *st8sia4* gene showing low mRNA levels in the brain, head kidney, gills, gonads and spleen^19^. Changes in the distribution of these genes compared to their human counterpart, and a weaker expression of the *st8sia4* gene further suggested that these fish genes likely define new subfamilies with novel and as yet unknown enzymatic specificities^19,24,25^. On the other hand, the orthologue of the *st8sia4* gene was lost in Neoteleosteii like the medaka *Oryzias latipes* and the orthologue of the *st8sia2* gene was lost in Siluriformes like *Ictalurus punctatus* and Esociformes like *Esox lucius*^19,26^. This further denoted evolution of these enzymes with potentially new functions for the remaining paralogue. The protein sequence of sialyltransferases is characterized by the presence of conserved sialylmotifs L, S, III and VS involved in substrates binding and in catalysis^22,27^. In addition, ST8Sia II and ST8Sia IV show unique conserved PolyBasic Region (PBR, 35 amino acids (aa)) and PolySialylTransferase Domain (PSTD, 32 aa) required for the selective recognition of NCAM and the catalytic activity of the human enzymes, respectively^28–30^. Preliminary sequence-based analysis using multiple sequence alignments and 3D-modeling of the PSTD unveiled potential changes of function in the salmonids polysialyltransferases^19^.

To better understand the molecular evolution of polysialyltransferases that potentially led to changes in substrate recognition and acquisition of new biological functions in human, each gene identified in the *C. maraena* genome (*Cma*ST8Sia II-r1, *Cma*ST8Sia II-r2 and *Cma*ST8Sia IV) was cloned and produced as a recombinant enzyme. Functional characterization of the salmonid enzymes highlighted unique catalytic specificities of *Cma*ST8Sia IV for the in vitro synthesis of various polymers of Sias and the exopolysialylation of the cell surface glycans.

## Results and discussion

### Molecular cloning, production and enzymatic activities of the Cma polysialyltransferases

**Figure 2:**
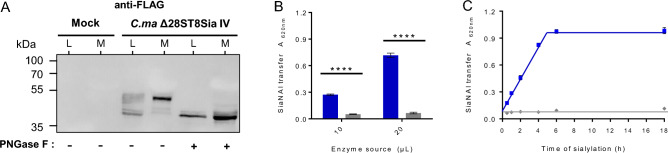
(**A**) Production of *Cma*ST8Sia IV in transfected HEK293 cells. HEK293 cells were transiently transfected with either an empty vector (Mock) or with the p3 × FLAG-CMV9-∆28ST8Sia IV (*CmaST8Sia IV*). Seven µg of proteins from each cell lysate (L) and 20 µL of each cell culture medium (M) with or without 50 U PNGase F treatment were separated on 8% SDS-PAGE and proteins were transferred on nitrocellulose membrane. WB was carried out with the anti-3 × FLAG antibody BioM2 (1 µg/mL). Original blot is presented in supplementary Fig. S9. Molecular weight markers are indicated on the left side. (**B**) Enzyme activity of recombinant *Cma*ST8Sia IV using the MPSA assay. A MPSA was carried out 4 h at 27 °C with 400 ng of the fish CD166/ALCAM, 100 µM CMP-SiaNAl and 10 or 20 µL of either the recombinant *Cma*ST8Sia IV (blue) or Mock (grey). Azido-PEG3-biotin was covalently attached to the alkyne group of transferred SiaNAl and biotin detected by the anti-biotin-HRP antibody. TMB (3,3′,5,5′-Tetramethylbenzidine) substrate was used and absorbance was measured at 620 nm^33^. Error bars in black represent SEM (n = 5), *p*-value < 0.0001. (**C**) Time course of sialylation by *Cma*ST8Sia IV in MPSA. Sialylation reactions were conducted at 27 °C using 400 ng of CD166/ALCAM and 100 µM CMP-SiaNAl using 20 µL of the fish enzyme *Cma*ST8Sia IV (blue squares) or of the Mock (grey diamonds) for 0.5, 1, 2, 4, 6 or 8 h (n = 2).

The deduced protein sequence of the newly identified *Cma*ST8Sia II-r1 and *Cma*ST8Sia II-r2 shared 63.8% and 62.7% sequence identity with their human homolog *Hsa*ST8Sia II, and *Cma*ST8Sia IV shared 78.8% sequence identity with the *Hsa*ST8Sia IV and all presented the four sialylmotifs and the polysialylmotifs PBR and PSTD characteristic of polysialyltransferases^28,31^. To achieve functional analyses, cDNA of *st8sia2r-1*, *st8sia2r-2* and *st8sia4* were PCR amplified from *C. maraena* gonads and brain, and soluble forms of the enzymes lacking their cytoplasmic and transmembrane domains were constructed in the p3 × FLAG-CMV9 expression vector. Since no good transfection yield could be achieved in the homologous salmonid fish cell line CHSE-214 system, secretion of the recombinant protein was optimized outside transiently transfected HEK293 cells, which remain the preferred host to produce difficult to express glycosyltransferases^32^. Cell lysates and culture medium collected 72 h post-transfection were run on SDS-PAGE and the recombinant *N*-terminally FLAG-tagged Δ28ST8Sia IV was detected with an anti-FLAG antibody both in the medium (M) and cell lysate (L) by Western Blot (WB) confirming a good level of protein production and secretion efficiency in this system (Fig. 2A; Supplementary Fig. S1). No signal could be detected with the empty plasmid transfected cells, and the relative expression and secretion levels of each fish ST8Sia II-related protein was low, as already described for the human glycoenzymes^32^. Several bands ranging from 40 to 52 kDa were detected in L and a major band around 52 kDa in M suggesting post-translational modification of the recombinant fish proteins. *N*-glycosidase F (PNGase F) treatment induced a shift to the expected molecular weight confirming the prediction (Fig. 2A; Supplementary Fig. S1).

Then, the enzymatic activities of these recombinant proteins were assessed with the newly set up Microplate Sialyltransferase Assay (MPSA) and the crude enzyme sources. The chemo-enzymatically synthesized CMP-SiaNAl reporter molecule (Fig. 1) was added as a substrate donor, as already described for the human ST6Gal-I and ST3Gal-I^33^. Since only a limited set of natural polysialylated proteins like NCAM have been described in the past^4^, we assayed a fish glycoprotein substrate that could serve as a suitable acceptor for these vertebrate polysialyltransferases, namely the fish CD166/ALCAM with sialylated bi-, tri- and tetra-antennae *N*-glycans (Supplementary Table S-1). This glycoprotein was coated on the microplate and sialylation reactions were carried out 4 h at 27 °C with each enzyme. Azido-PEG3-biotin was then covalently attached to the alkyne group of transferred SiaNAl units and biotin was detected using an HRP-conjugated anti-biotin antibody. As shown in Fig. 2B, significant and reproducible data were obtained on both acceptors showing that the fish ST8Sia IV is active, stable and efficiently transfers SiaNAl from CMP-SiaNAl. Initial velocity was proportional to the enzyme amount up to 20 µL and sialylated product appearance was linear up to 4 h of sialylation (Fig. 2C). However, the fish ST8Sia II-r1 and ST8Sia II-r2 demonstrated no chemoenzymatic modification of these substrates under these conditions (Supplementary Fig. S1). This is in line with a previous study by Kitajima’s group reporting very low levels of enzymatic activity of recombinant polysialyltransferases from *O. mykiss*^24^. Although still speculative, this observation also corroborates the previously reported inhibition of the human ST8Sia II using a propyl-C5 modified Sia precursor in vivo^34^.

**Figure 3:**
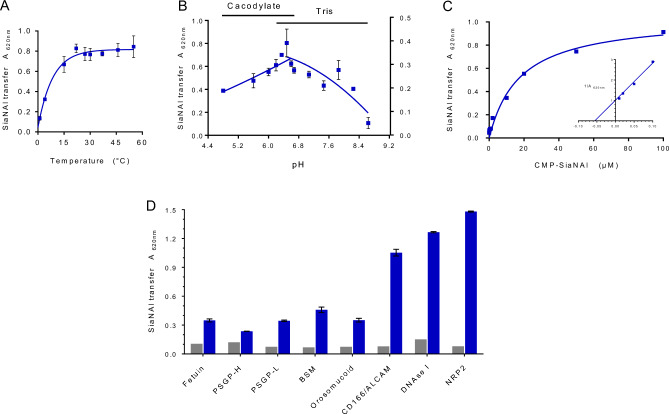
Optimization of sialylation conditions in the MPSA. (**A**) Effect of temperature on the sialylation by *Cma*ST8Sia IV. Sialylation was performed 4 h on 400 ng of CD166/ALCAM with 100 µM CMP-SiaNAl and 20 µL of *Cma*ST8Sia IV at different temperatures from 0 °C to 60 °C (n = 2). (**B**) Effect of pH on the sialylation by *Cma*ST8Sia IV. Sialylation reactions were performed 4 h at 27 °C on 400 ng of CD166/ALCAM with 100 µM CMP-SiaNAl and 20 µL of *Cma*ST8Sia IV at different pH from 4.8 to 8.6 (n = 2). (**C**) Determination of kinetic parameters of ST8Sia IV towards donor substrates. Sialylation reactions were carried out in cacodylate buffer pH 6.4, at 27 °C for 4 h with 20 µL of fish ST8Sia IV. CMP-SiaNAl was used at various concentrations ranging from 0 to 100 μM with 400 ng of CD166/ALCAM (n = 3). Michaelis–Menten constants were determined graphically with GraphPad. *Cma*ST8Sia IV shows an apparent Km of 17.60 + / − 2.52 µM towards donor substrate. (**D**) Enzymatic activities on several acceptors in the MPSA. Sialylation reactions were performed 4 h at 27 °C using 400 ng of various acceptors (fetuin, PSGP-H, PSGP-L, BSM, orosomucoid, CD166/ALCAM, DNase I and NRP2) with 100 µM of CMP-SiaNAl and with 20 µL of *Cma*ST8Sia IV (blue) or Mock (grey). Error bars represent SEM (n = 2).

Therefore, we focused on the fish ST8Sia IV to assess the robustness of the enzyme activity and set up optimum polysialylation conditions, the influence of pH (4.8–8.6, measured at 27 °C) and temperature (0–60 °C) was studied on enzyme velocity. An optimal temperature of 27 °C was found for the fish ST8Sia IV, which remained highly active at high temperatures (Fig. 3A) suggesting that during evolution, fish sialyltransferases could have acquired a higher level of structural flexibility to adapt to variable environment^35^. Our data demonstrated a conserved optimum pH around 6.4 for this fish polysialyltransferase (Fig. 3B), comparable to those determined in another enzymatic assay for the human ST8Sia II^36^. Using assay conditions set at pH 6.4, 27 °C, kinetic parameters of the recombinant fish enzyme towards CMP-SiaNAl were calculated. An apparent Michaelis constant (Km) value of 17.60 ± 2.52 µM was calculated for fish ST8Sia IV according to the Michaelis–Menten model (Fig. 3C). This value is in the same order of magnitude as found for the human ST6Gal I and ST3Gal I towards CMP-SiaNAl^33^. To assess the enzyme specificity and the acceptor substrate preference of the fish ST8Sia IV, several glycoproteins with various *N*- and *O*-glycan structures (Supplementary Table S-1) were used as acceptors and coated on microplates for sialylation assays. *Cma*ST8Sia IV was active transferring SiaNAl on bovine fetuin, fish CD166/ALCAM, bovine submaxillary mucin (BSM), human DNase I, human NRP2, fish polysialoglycoprotein PSGP-H, PSGP-L and human orosomucoid (α(1)-acid glycoprotein) (Fig. 3D).

### Structural assessment of polySias generated by the Cma polysialyltransferases

**Figure 4:**
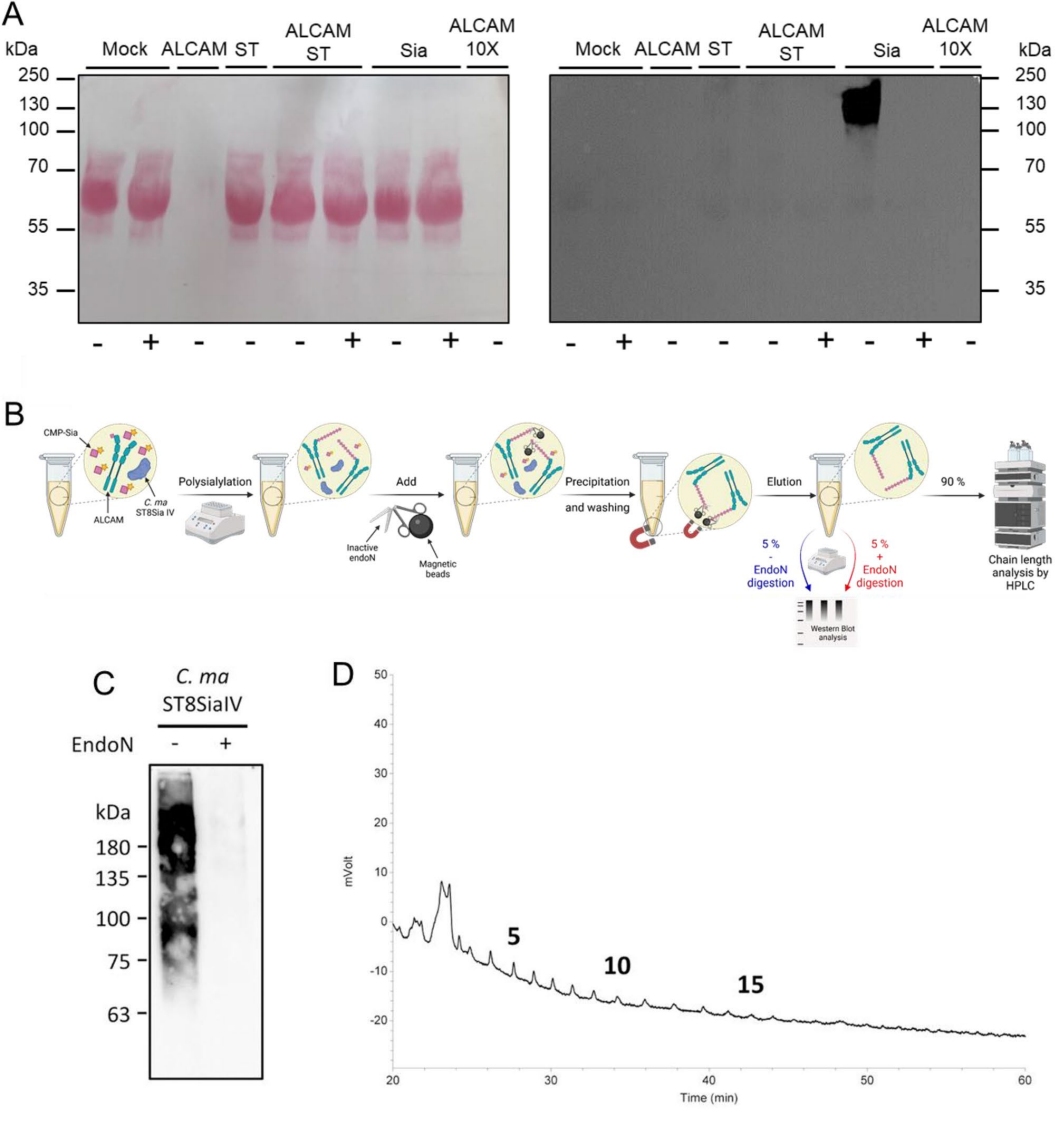
Chain length analysis of CD166/ALCAM after polysialylation with CMP-Neu5Ac by *Cma*ST8Sia IV. (**A**) Prior to sialylation, no polySia can be detected by the mAb735 on the various actors of the sialylation reaction *i.e.* CD166/ALCAM (ALCAM) or concentrated ALCAM (ALCAM 10 ×), HEK293 cell culture medium (Mock) or *Cma*ST8Sia IV (ST). PolySia is detected only after 4 h of polysialylation of CD166/ALCAM with the *Cma*ST8Sia IV (Sia). Original blot is presented in supplementary Fig. S9 (**B**) Illustration of the precipitation-workflow to enrich polySia for HPLC analysis using inactive endoN. Created with BioRender.com. (**C**) An aliquot (5% per lane) of the eluate was used for WB to monitor the polysialylation and precipitation**.** Original blot is presented in supplementary Fig. S9 (**D**) The remaining precipitate (90%) was used for “mild” DMB labeling. Resulting fluorescently labeled sialic acid polymers were separated via anion-exchange chromatography. The chain length is given for selected peaks.

To explore the polysialyltransferases’ ability to use natural CMP-Sia donors and successively add several Sia residues, we achieved sialylation reaction and detected polysialylated products after SDS-PAGE and WB with the mAb735 antibody, which recognizes polyNeu5Ac with a DP ≥ 8 residues^37^. We firstly checked that no pre-existing polySia could be detected with the mAb735 on the various reagents prior sialylation reaction (Fig. 4A). Then, sialylation reactions were performed with the fish ST8Sia IV and its human orthologue, 100 µM of chemo-enzymatically synthesized CMP-Neu5Ac (Fig. 1) and 6 µg of CD166/ALCAM for 0.5 to 8 h (Supplementary Fig. S2). Within 30 min, an extensive smear ranging from 70 to more than 250 kDa could be detected confirming that both the human and fish ST8Sia IV could synthetize polyNeu5Ac chains. In addition, we verified the polysialylation activity of fish ST8Sia IV by HPLC analysis. As a first step, polysialylated CD166/ALCAM was enriched with an enzymatically inactive form of the endoneuraminidase N (endoN) coupled to magnetic beads. This is possible because endoN contains an oligo/polySia-binding domain^38^. The polysialylation and affinity-precipitation were monitored by WB against polySia using an aliquot of the eluate (5%) (Fig. 4B and C). The remaining sample (90%) was analyzed by a HPLC approach to determine the chain length. To this end, polySia chains were released and labeled with 1,2-diamino-4,5-methylendioxy-benzene (DMB). The resulting fluorescently labeled polymers were separated according to their DP by anion exchange chromatography. In line with the WB results, polySia chains consisting of more than 7 Sias were observed (Fig. 4D). Thus, the polysialylation activity of fish ST8Sia IV was verified by two independent methods.

**Figure 5:**
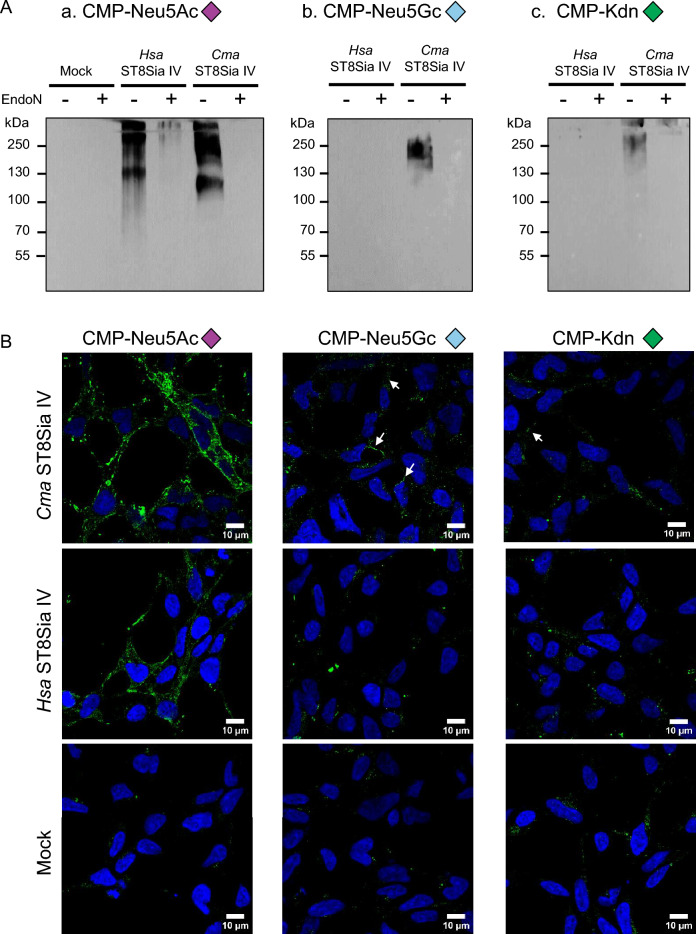
CD166/ALCAM polysialylation achieved with human and *Cma* polysialyltransferases and different CMP-sialic acids. (**A**) Polysialylation with ST8Sia IV enzymes visualized on WB with the mAb735**.** Sialylation reactions were performed 4 h at 27 °C with 100 µM of CMP-Neu5Ac, CMP-Neu5Gc or CMP-Kdn (purple, light blue and green diamonds, respectively) on 6 µg of CD166/ALCAM with 150 ng of *Hsa*ST8Sia IV or 20 µL of either *Cma*Δ28ST8Sia IV or Mock. Treatment with endoN for 1 h at RT was used as a control to remove polysialylation. Sialylation products were heated 5 min at 60 °C in Laemmli buffer, separated on 6% SDS-PAGE and transferred onto PVDF membranes. Blots were probed with mAb735 (0.5 µg/mL). Original blots are presented in supplementary Fig. S9. Molecular weight markers are indicated on the left side. Immunoblots were incubated with femtoECL and detected after 5 s exposure on for a) CMP-Neu5Ac, 30 s for b) CMP-Neu5Gc and 180 s for c) CMP-Kdn panels. (**B**) Exosialylation of HEK293 cells with *Hsa*ST8Sia IV and *Cma*ST8Sia IV**.** After EndoN treatment, sialylation reactions were carried out 4 h at 27 °C on fixed cells with 40 µL *Cma*ST8Sia IV or 200 ng of the *Hsa*ST8Sia IV or 40 µL of the Mock and 500 µM of donor substrates: a) CMP-Neu5Ac, b) CMP-Neu5Gc and c) CMP-Kdn. PolySia on cell surface was detected by mAb735 (green), nuclei were stained with DAPI (blue). Fluorescence was detected using a Carl Zeiss confocal microscope. Scale bars: 10 µm (Zoom 2 ×). White arrows indicate polySia formed by *Cma*ST8Sia IV with CMP-Neu5Gc and CMP-Kdn.

To evaluate the fish and human ST8Sia IV ability to use various Sia donors, sialylation reactions were performed in cell-free or cell-based assays with 100 µM natural CMP-Sia. Firstly, to avoid background and sialylation inhibitor side products due to CMP-Sia hydrolysis, we chemo-enzymatically synthesized various natural donor substrates CMP-Neu5Ac, CMP-Neu5Gc and CMP-Kdn (Fig. 1) using either the CMP-Sia synthetase (CSS) from *Neisseria meningitidis*^39^ or the recently described rainbow trout CMP-Kdn synthetase (rtCSS)^40^ and checked efficiency of the reaction by ^31^P NMR (Supplementary Fig. S3). After sialylation reaction using CD166/ALCAM as an acceptor for polySia chains, sialylated products were run on SDS-PAGE, transferred on nitrocellulose membranes and detected with the mAb735. In addition, endoN treatment was used as an additional control to remove the newly formed polySias with a DP ≥ 8^38^. Broad signals between 100 and 250 kDa were visualized as expected for both enzymes with CMP-Neu5Ac on WB using the mAb735 (Fig. 5Aa). Remarkably, a smear of polySia could be detected to variable extents with the mAb735 after polysialylation with CMP-Neu5Gc (Fig. 5Ab) or CMP-Kdn (Fig. 5Ac) and the *Cma*ST8Sia IV, which could be removed by endoN treatment (Fig. 5A). In contrast to fish ST8Sia IV, no polySia could be detected after polysialylation with CMP-Neu5Gc (Fig. 5Ab) or CMP-Kdn (Fig. 5Ac) by the human polysialyltransferase denoting distinct substrate specificities between each orthologue.

Comparable data were obtained with a cell-based approach using the human and fish enzymes to selectively introduce various natural Sia donors on cell surface glycoconjugates of HEK293 cells (Fig. 5B). Prior to exogenous polysialylation, we ensured that HEK293 cells displayed NCAM and checked its polysialylation status (Supplementary Fig. S4). Then, polySia of paraformaldehyde fixed HEK293 cells was degraded by endoN. After exogenous polysialylation, polySia could be detected with mAb735 using CMP-Neu5Ac for both enzymes, whereas lower levels of polySia could be detected using CMP-Neu5Gc only with the *Cma*ST8Sia IV, but not with the *Hsa*ST8Sia IV (Supplementary Figs. S5–S7). No conclusive results could be obtained after exo-polysialylation with CMP-Kdn and the fish, human or mock enzyme sources (Fig. 5B).

**Figure 6:**
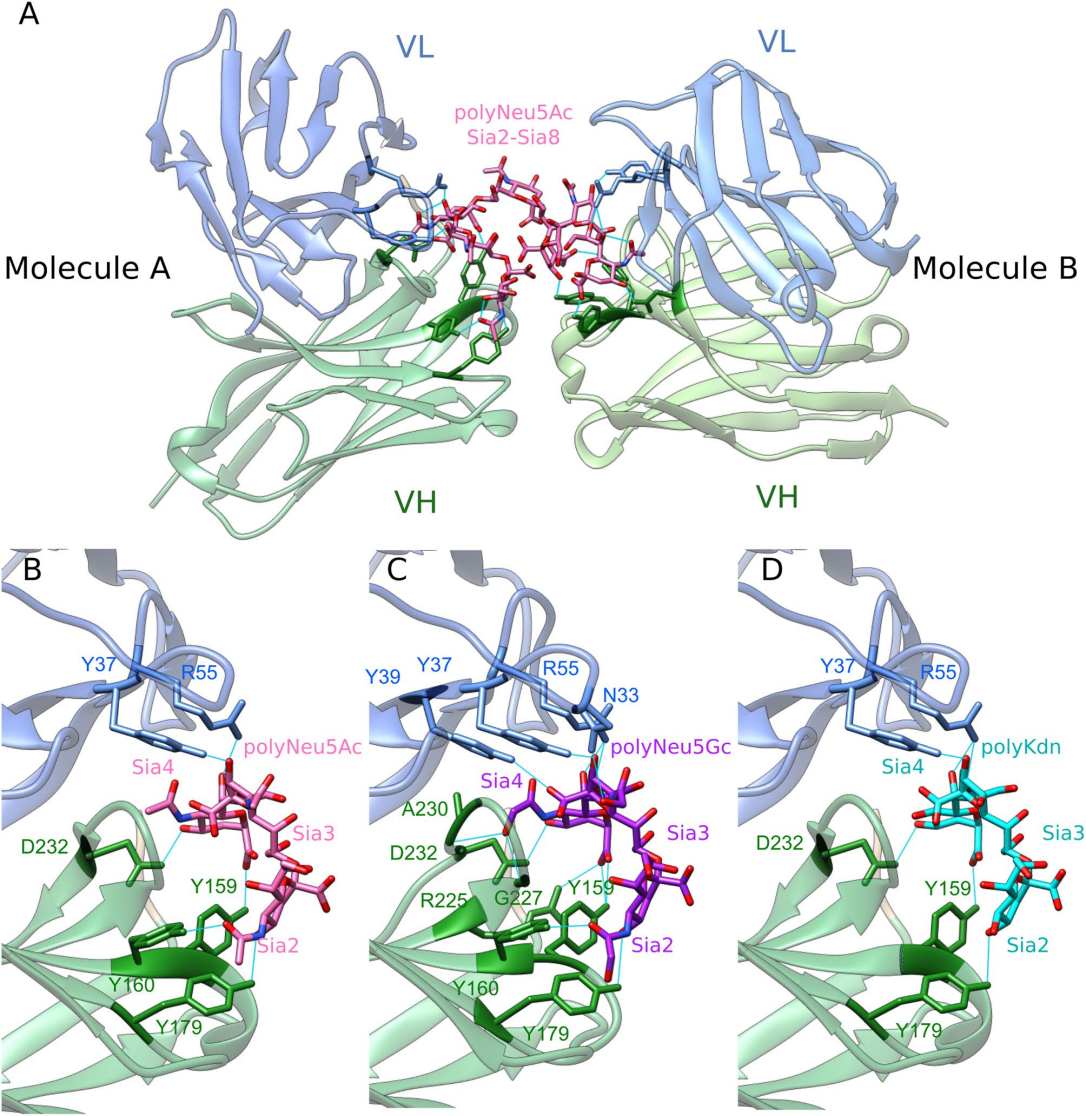
Crystal structure of scFv735 (PDB 3WBD) in complex with α2,8-linked octasialic acids. (**A**) Overall crystal structure of scFv735 in complex with polyNeu5Ac, variable light domain (V_L_) and variable heavy domain (V_H_) are coloured in blue and green, respectively. The Sia residues interacting with two scFv735 molecules are highlighted in pink. Hydrogen bonds between Sia and the antibody are shown in cyan. Residues involved in direct hydrogen bonds are shown in stick representation. Close-up view of the antigen recognition site of scFv735 in complex with trisialic acid of (**B**) Neu5Ac, (**C**) Neu5Gc and (**D**) Kdn.

As our experimental data pointed to the ability of mAb735 to detect polyNeu5Gc and polyKdn, and endoN to cleave all polySias, we further checked the reliability of each polymer interactions with mAb735 through computational modeling. A previous study reported crystallization of the single chain Fv fragment of mAb735 (scFv735) in complex with an octasialic acid polymer of α2,8-linked Neu5Ac (PDB entry 3WBD)^37^. In this complex, two antibody molecules (A and B) facing each other are associated with an octasialic acid. As illustrated in Fig. 6A, one scFv735 molecule (B) interacts with Sia2–Sia4, whereas the other (A) interacts with Sia6 –Sia8. The antigen-binding site is comprised of six complementarity-determining regions (CDR) L1, L2, L3 and H1, H2, and H3 interacting with three consecutive Sias through direct and indirect hydrogen bonds. Six amino acids are involved in direct hydrogen bonds; four of them Tyr37, Arg55, Tyr159 and Asp232 are critical for antigen recognition, whereas Tyr160 and Tyr179 are less important. In addition, indirect interactions involve 11 structured water molecules. To investigate to what extent analogous interactions could be found between the antibody and polySias made of eight Neu5Gc or Kdn, we modified the Neu5Ac to Neu5Gc/Kdn molecules in the PDB 3WBD. We showed that in addition to the six direct hydrogen bond interactions described for polyNeu5Ac (Fig. 6B), five others could be established between Tyr39, Ala230, Gly227, Arg225 and Asn33 for polyNeu5Gc (Fig. 6C), whereas five out of the six aa critical for polyNeu5Ac recognition were retrieved for polyKdn (Fig. 6D, Supplementary Fig. S8) supporting our experimental data. Altogether, we provided here for the first time, computational and experimental evidences for the recognition to various levels of various polySia chains using the mAb735.

**Figure 7:**
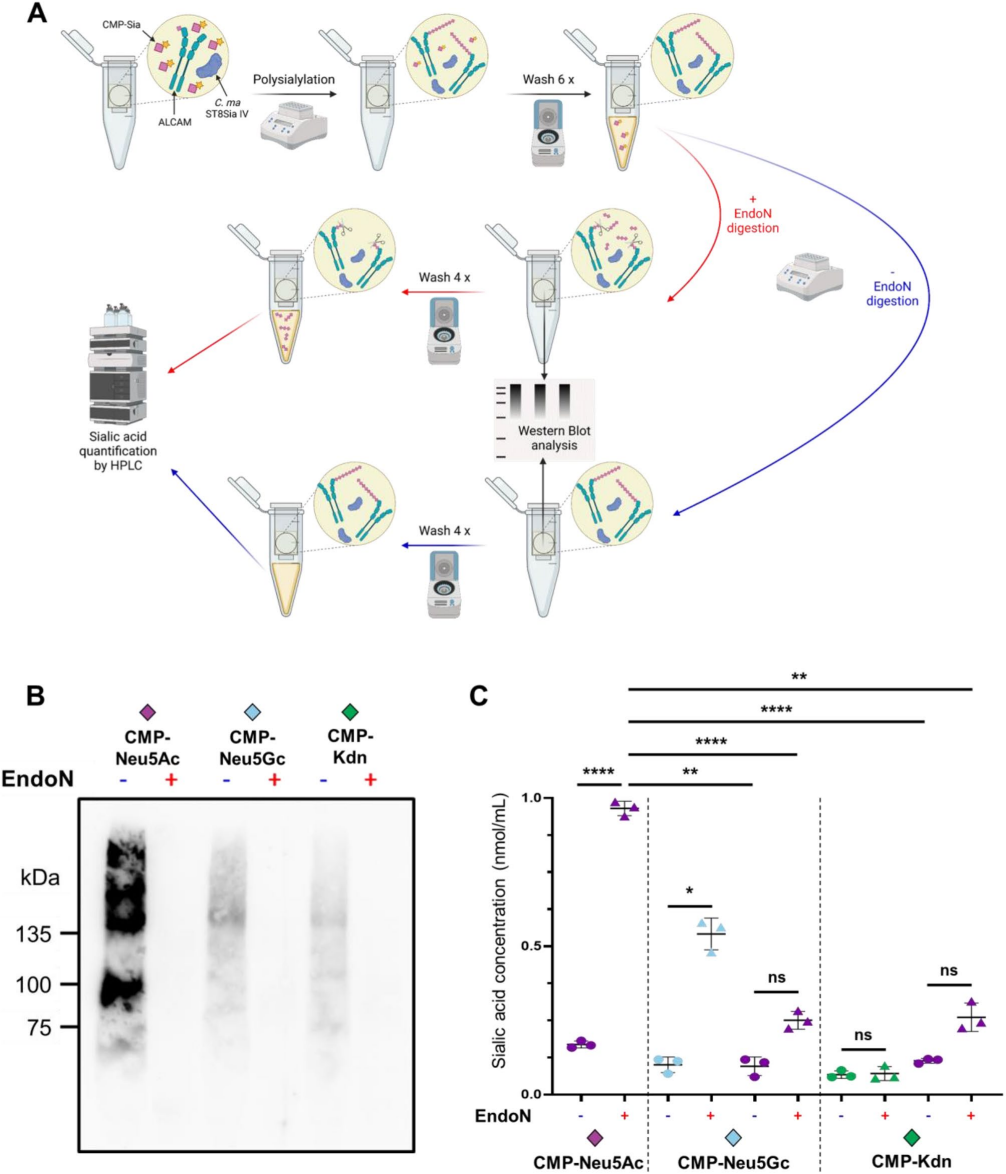
Composition analysis of polySia on CD166/ALCAM. (**A**) Workflow of the centrifugal filter polysialylation assay to quantitatively analyze the transfer of different Sia residues to CD166/ALCAM by *Cma*ST8Sia IV. Created with BioRender.com. (**B**) The polysialylation reaction using different CMP-Sia (CMP-Neu5Ac, CMP-Neu5Gc or CMP-Kdn) was controled by WB using the mAb735. Original blot is presented in supplementary Fig. S9. (**C**) In the final flow-throughs of untreated (circle) and endoN treated (triangle) samples, sialic acids were analyzed by HPLC for each experimental set-up: donor substrate CMP-Neu5Ac, quantification of Neu5Ac (purple); donor substrate CMP-Neu5Gc: quantification of Neu5Gc (blue) and Neu5Ac (purple); donor substrate CMP-Kdn, quantification of Kdn (green) and Neu5Ac (purple). The means and standard deviations of three independent experiments are displayed. Statistical differences are indicated: *p < 0.05; **p < 0.01; ****p < 0.0001; *ns* not significant.

To check the transfer of Neu5Ac, Neu5Gc and Kdn by *Cma*ST8Sia IV with an antibody independent approach, a HPLC-based strategy was applied (Fig. 7A). To this end, polysialylation of CD166/ALCAM was performed in centrifugal filter units. After polysialylation, remaining CMP-Sia was removed before polySia degradation by endoN. Untreated samples (without endoN treatment) were used as negative control. Polysialylation as well as degradation by endoN were successfully performed in the centrifugal filter units as checked by WB (Fig. 7B). After a final filtration step, the flow-throughs were analyzed by HPLC. Newly added Sia residues could be determined comparing the Sia content in the flow-through of untreated and endoN treated samples. When CMP-Neu5Ac was used as substrate, a strong increase of Neu5Ac was detected after endoN digestion indicating that *Cma*ST8Sia IV efficiently transfered Neu5Ac to CD166/ALCAM (Fig. 7C). When CMP-Neu5Gc was used, an increase of Neu5Gc was also observed although lower than that obtained with CMP-Neu5Ac, which is in line with the WB and the cell-based data obtained. However, the transfer of several Kdn residues could not be proven since the Kdn values of the endoN and the untreated sample were comparable. Since slightly higher amounts, but not statistically significant amounts of Neu5Ac were detectable in endoN treated samples of the CMP-Neu5Gc and CMP-Kdn setups (Fig. 7C), it is proposed that pre-existing oligo-Neu5Ac are degraded by endoN, which were not detectable by mAb735. The elongation of already existing Neu5Ac-oligomers using CMP-Kdn let sugest that the transfer of a few Kdn residues was likely not sufficient to be detected by HPLC, but enabled binding of mAb735, a hypothesis that is supported by the WB data (Fig. 5). Altogether, these results demonstrated that CMP-Neu5Ac is the preferred substrate donor of *Cma*ST8Sia IV; however, in constrast to the human enzyme, this fish ST8Sia IV enzyme does also accommodate CMP-Neu5Gc and to a lesser extent CMP-Kdn.

In summary, this study highlights original enzymatic activities and specificities of the salmonid ST8Sia IV likely responsible for the synthesis of diverse polySias. Fish polysialyltransferases show functional divergence acquired during vertebrate evolution with specific characteristics and kinetic properties distinct from those of human enzymes. Since no crystal 3D-structure of vertebrate polysialyltransferase has been obtained yet, molecular phylogeny, modeling and docking strategies will be developed to shed light on the molecular basis of these differential substrate recognition by vertebrate polysialyltransferases and better understand their biological function. In addition, our data brought new evidences of the usefulness of the molecular tools mAb735 and endoN towards a variety of natural polySia polymers.

## Materials and methods

### Materials

Fetuin, orosomucoid, bovine submaxillary mucin (BSM), 3,3',5,5'-tetramethylbenzidine (TMB), azide-PEG3-biotin, monoclonal Anti-FLAG^®^ BioM2 antibody (1 mg/mL), sialic acid aldolase from *Escherichia* *coli* K-12, CSS from *Neisseria* *meninigitidis*, pyrophosphatase from *Saccharomyces* *cerevisiae* (PPase), Neu5Ac, Neu5Gc and Kdn were purchased from Sigma-Aldrich (Saint Quentin Fallavier, France). Sodium cytidine-5′-triphosphate (CTP) was obtained from TCI chemicals. Human Neuropilin-2 (NRP2) was purchased from Interchim. Horseradish peroxidase (HRP) conjugated anti-biotin antibody (monoclonal fraction of mouse IgG; 0.8 mg/mL) was purchased from Jackson Immunoresearch. 2-[4-((bis[(1-tert-butyl-1H-1,2,3-triazol-4-yl)methyl]amino)methyl)-1H-1,2,3-triazol-1-yl]acetic acid (BTTAA) was synthesized in our laboratory as described previously^33^. The human recombinant enzymes Δ23ST8Sia II and Δ39ST8Sia IV were from R&D Systems (Rennes, France). Human Recombinant Deoxyribonuclease I (DNase I) was purchased from Boster Bio (Pleasanton, CA, USA). PageRuler Plus Prestained Protein Ladder was from Thermo Scientific. Anti-polySia mAb735 (Rabbit IgGκ, 1 mg/mL) was obtained from Enzo Life Sciences. Sodium cacodylate buffer was from Prolabo (Paris, France).

Whitefish *C. maraena* were provided by the Institute of Fisheries of the Mecklenburg Western Pomerania Research Center for Agriculture and Fisheries (Born, Germany). The present study exclusively utilised samples from previous experiments^41,42^ following the 3R principles in science. These experiments had been approved by the Landesamt für Landwirtschaft, Lebensmittelsicherheit und Fischerei, Mecklenburg‐Vorpommern, according to the German animal welfare law (approval ID: LALLF M‐V/TSD/7221.3‐1‐069/18; November 2018) and all the methods were carried out in accordance with relevant Institutional guidelines and regulations and with the ARRIVE guidelines. Brain and kidney tissues from whitefish were snap frozen in liquid nitrogen and kept at − 80 °C for RNA isolation.

### Bioinformatics analyses of polysialyltransferases sequences and molecular docking

Nucleotide sequences of *st8Sia2r-1/st8sia2a* (NCBI nucleotide accession code: XM_041891035), *st8sia2r-2/st8sia2b* (XM_041836579) and *st8sia4* (XM_041897387) genes from closely related *Coregonus clupeaformis* were synchronised with the transcriptome of *Coregonus maraena* (Bioproject ID: 302,355) previously^19^ and compared with the orthologous sequences in human ST8SIA2 (NM_006011) and ST8SIA4 (NM_005668).

Multiple sequence alignments of the corresponding protein sequences were achieved using ClustalW at PRABI-Gerland (https://npsa-prabi.ibcp.fr/cgi-bin/npsa_automat.pl?page=/NPSA/npsa_clustalw.html). The amino acid sequence analysis was performed using the software of Expert Protein Analysis System (ExPASy; Swiss Institute of Bioinformatics, Switzerland; website (https://www.expasy.org/, last accessed on: 04 November 2022). Hydropathy analyses and determination of potential N-glycosylation sites were performed using the servers TMHMM—2.0: Prediction of Transmembrane Regions (https://services.healthtech.dtu.dk/service.php?TMHMM-2.0, last accessed on: 04 November 2022) and the NetNGlyc 1.0 Internet program (https://services.healthtech.dtu.dk/service.php?NetNGlyc-1.0, last accessed on: 04 November 2022) of ExPASy. Sequences were also submitted to Compute pI/MW (ExPASy) analysis to determine their theoretical molecular weight.

To investigate to what extent analogous interactions could be found between the antibody and polySia chains made of eight Neu5Gc or Kdn, we took as reference direct and indirect interactions described for mAb735 complexed with polyNeu5Ac in ProteinDataBank (PDB) 3WBD and modified the Neu5Ac to Neu5Gc/Kdn molecules in the PDB 3WBD using the biocomputing software UCSF Chimera. The complex was minimized using the AMBER force field, and charges were computed using ANTECHAMBER^43^.

### Construction of Cma polysialyltransferases expression vectors

Total RNA was extracted from homogenized brain or kidney tissue using the Nucleospin RNA Plus (Macherey–Nagel, Düren, Germany) and quantified with the NanoDrop ^®^ND-1000 spectrophotometer (NanoDrop Technologies, Wilmington, DE, USA). Total RNA was reverse-transcribed using the Maxima First strand cDNA synthesis for RT-qPCR kit according to the manufacturer protocol (Thermo Scientific). To obtain a full-length cDNA, an initial RT-PCR was performed with each kidney and brain cDNA preparation, Q5 high fidelity DNA polymerase (New England Biolabs), sense and antisense oligonucleotide primers containing *Hind*III and *Kpn*I restriction sites respectively (listed in Table S-2). PCR reactions were run for 2 min at 98 °C followed by 35 cycles (98 °C for 30 s, 65 °C for 30 s, 72 °C for 15 s) and an extension step of 2 min at 72 °C. The resulting 1074 bp amplified *st8sia4* cDNA was subcloned into the pJET vector of Clone JET PCR cloning 1.2 kit (Thermo Scientific), cut out with *Hind*III and *Kpn*I and inserted into the *Hind*III and *Kpn*I sites of the plasmid p3 × FLAG-CMV10 expression vector. The resulting 1075 bp amplified *st8sia2r*-1 cDNA was inserted directly into p3 × FLAG-CMV10. For s*t8sia2*r-2, a synthetic vector based on its full-length sequence was constructed using the pCDNA3.1 plasmid at GeneArt Instant Designer (ID project: 2020AAEFFD, Thermo Fisher Scientific). A cDNA encoding a truncated form of the *Cmast8sia2r*-1, *Cmast8sia2r*-2 and *Cmast8sia4* lacking their first 35, 35 or 28 aa residues of the open reading frame, respectively were amplified by PCR using the previous construct p3 × FLAG-CMV10 for *st8sia4* and *st8sia2*r-1 and pcDNA3.1 for *st8sia2*r-2 as templates. Sense and antisense primers are described in Supplementary Table S-2. Touchdown reactions were run for 2 min at 94 °C followed by 10 cycles (94 °C for 30 s, 60 °C (minus 1 °C per cycle) for 30 s, 72 °C for 70 s) and then 20 cycles (94 °C for 30 s, 55 °C for 30 s, 72 °C for 70 s) and an extension step of 5 min at 72 °C. The resulting PCR amplified cDNAs were inserted into the *Hind*III and *Kpn*I sites of the p3 × FLAG-CMV9 expression vector. These last constructs encoded a FLAG-tagged protein with a signal peptide sequence, the FLAG octapeptide and the three ∆35ST8Sia II-r1, ∆35ST8Sia II-r2 and ∆28ST8Sia IV sequences deleted of their cytoplasmic tail and transmembrane domain.

### Cell culture, Transfection and Exosialylation of HEK293 surface using the human and fish ST8Sia IV and confocal microscopy

HEK293 cells (ATCC CRL-1573) and CHO-K1 (ATCC CCL-61) cells were grown in Dulbecco’s modified eagle’s medium (DMEM, Dutscher) supplemented with fetal calf serum (10% FCS, Biowest) at 37 °C, 5% CO_2_. Confluent cells (~ 70%) were transiently transfected with either 2 or 10 μg of purified expression constructs in 6 well plates or in 100 mm petri dishes with Lipofectamine 2000 (Thermo Scientific) in UltraMEM medium (12-743F, Lonza) according to the manufacturer's instructions. An empty p3 × FLAG-CMV9 plasmid was used as a control (Mock). Transiently transfected cells and cell culture media were collected 72 h after transfection. Recombinant polysialyltransferases produced in DMEM-FCS culture media were used as a crude enzyme source for enzymatic assays. The p3 × FLAG-CD166/ALCAM plasmid was transiently transfected in CHO-K1 cells and the recombinant protein was secreted in the UltraMEM medium. This medium was collected 48 h after transfection and centrifuged 30 min at 10,000 *rpm* and at 4 °C to remove cell debris. The CD166/ALCAM content of the UltraMEM medium of the p3 × FLAG-CD166/ALCAM or mock transfected CHO-K1 cells was assessed with the micro-BCA protein assay kit (23,235, Thermo Scientific) and WB with the anti-FLAG (see below).

For the exosialylation experiments, HEK293 cells were grown on glass coverslips for 24 h, then washed three times with PBS and fixed in 4% PFA for 30 min at room temperature (RT). After three washes with PBS, cells were treated with endoN at 100 ng/mL for 1 h at 37 °C. Excess of endoN was eliminated by five PBS washes 5 min under gently agitation. Sialylation reactions were performed at 27 °C for 4 h with the chosen enzymatic source of enzyme and 500 µM of CMP-Sia donor substrate in 100 µL final volume. The rest of the experiment consists of immunofluorescence to visualize de novo sialylation. Briefly, after three washes with PBS, the cells were incubated with blocking buffer (2% BSA in PBS) for 1 h at RT and then overnight at 4 °C in humid chamber with the primary antibodies diluted in blocking buffer: 2 µg/mL anti-polySia mAb735 (Enzo Lifes Sciences) and 5 µg/mL anti-CD56 (NCAM, clone 123C3) (Invitrogen, ThermoFisher Scientific). After three washes with PBS, cells were incubated 1 h at RT in the dark with AlexaFluorTM conjugated secondary antibodies: respectively goat anti-rabbit 488 and goat anti-mouse 568 (Invitrogen, ThermoFisher Scientific) diluted at 1: 600 (3.33 µg/mL) in blocking buffer. Finally, cells were washed three time with PBS, the nuclei were stained with DAPI (5 µg/mL in PBS) and the coverslips were mounted on glass slides with Mowiol^®^. The fluorescence was detected using the inverted Zeiss LSM780 confocal microscope with a 40 × oil immersion and data were collected with the ZEN 2010 software (Zeiss, Oberkochen, Germany). The images were analyzed with ImageJ software.

### Western blot analyses

WB was used to visualize and quantify tagged recombinant proteins secreted from transiently transfected HEK293 or CHO-K1 cells. For that purpose, 20 μL of culture media of transfected cells were boiled 5 min at 95 °C in 4 × Laemmli buffer (235 mM Tris–HCl pH 6.8, 8% SDS, 40% glycerol, 10% β-mercaptoethanol, 0.01% bromophenol blue), and resolved by 8% SDS-PAGE. Proteins were transferred onto Amersham Protran nitrocellulose membrane (GE Healthcare Life Sciences) for 75 min at 200 mA and checked with Ponceau red staining (5% acetic acid, 0.1% Ponceau). Membranes were washed, then blocked using 5% non-fat milk in TBS-T (TBS with 0.05% Tween 20). Detection of recombinant proteins was achieved with the primary mouse anti-FLAG^®^ M2 antibody (1 µg/ml; Sigma-Aldrich) in TBS-T 0.05% overnight at 4 °C. After 3 washes with TBS-T 0.05%, the membrane was incubated with an anti-mouse antibody coupled to horseradish peroxidase (HRP) (0.1 µg/mL; InVitrogen) for 1 h then was washed 5 times with TBS-T 0.05%. Blots were developed using enhanced chemiluminescence (ECL West Pico Plus or ECL West Femto, Thermo Scientific). The images were acquired using a CCD camera (Fusion Solo, Vilber Lourmat) and the Fusion software. Quantification and densitometry analysis were done with ImageJ and GraphPad Prism 6.

WB strategy was also used after polysialylation reaction achieved on CD166/ALCAM with the natural substrate donors (see below). Polysialylated CD166/ALCAM products treated or not with endoN were heated 5 min at 60 °C in 4 × Laemmli buffer, separated on an 6% SDS-PAGE gel for 90 min at 95 V (1 × Tris–Glycine buffer (Euromedex)/ 20% Methanol) then transferred onto nitrocellulose membrane. Membranes were saturated in 5% non-fat milk in TBS-T 0.05% for 1 h at room temperature (RT) and incubated with the anti-polySia mAb735 antibody (1 µg/mL) or endoN (6.7 µg/mL) overnight at 4 °C. After 3 washes with TBS-T 0.05%, the membrane was incubated with secondary anti-rabbit antibody coupled to HRP (0.1 µg/mL; InVitrogen) for 1 h and was washed 5 times with TBS-T 0.05%. Detection was achieved by chemiluminescence as reported above.

### Chemo-enzymatic synthesis of activated CMP-sialic acids and sialylation assays

*N*-4-pentynoylneuraminic acid (SiaNAl) and the various natural CMP-Sia were synthetized as previously described^39^. In brief, *N*-mannosamine was converted to *N*-4-pentynoylmannosamine by coupling with succinimidyl 4-pentynoate, which was then reacted with sodium pyruvate in the presence of sialic acid aldolase from *E. coli* K12 in pH 7.5 phosphate buffer and purified by anion exchange chromatography (Dowex 1 × 8) and gel filtration chromatography (P2 resin) to yield pure SiaNAl (86% overall yield over 2 steps).

The syntheses of cytidine-5′-monophospho-N-sialic acids (CMP-Sia) CMP-Neu5Ac, CMP-Neu5Gc, CMP-Kdn and CMP-SiaNAl were carried out in equimolarity of CTP and each Sia (1:1) with 0.3 U CSS from *N. meningitidis*^39^ or the rainbow trout CMP-Kdn synthetase (rtCSS)^40^ and 0.5 U PPase in 100 mM Tris–HCl, 20 mM MgCl_2_ buffer (pH 8.5) at 37 °C. The reactions were carried out in a 5 mm NMR tube and monitored by ^31^P NMR spectroscopy in a Brüker Avance II 400 MHz spectrometer (Supplementary Fig. S2). Upon completion, the pure formed CMP-Sia was immediately stored at − 80 °C until use^33,39^.

Enzymatic assays were performed using the MicroPlate Sialyltransferase Assay (MPSA) as described previously^33^. In brief, 400 ng of glycoprotein acceptors in 100 μL of sodium bicarbonate buffer (20 mM, pH 9.6) were adsorbed into the bottom of 96-well plates (F8 MaxiSorp Loose Nunc-Immuno Module ThermoScientific) at 4 °C overnight. After three washes with 150 μL of phosphate buffered saline containing 0.05% Tween 20 (PBS-T 0.05%), saturation was carried out for 1 h at RT with 100 μL of oxidized BSA at 0.05% diluted in bicarbonate buffer. The sialylation transfer reaction was carried out for one to several hours at 27 °C with the chosen enzymatic source and 100 μM of CMP-SiaNAl in 100 mM cacodylate buffer (MnCl_2_ 10 mM, Triton CF-54 0.2%, pH 6.2) in a final volume of 100 μL. After sialylation, the wells were washed with PBS-T 0.05%, then the CuAAC labeling reaction was performed by adding 100 μL of a solution containing 300 μM CuSO_4_, 600 μM BTTAA, 2.5 mM sodium ascorbate and 250 μM azide-PEG3-biotin in PBS^44^. After 1 h at 37 °C, the reaction was stopped by washing three times with PBS-T 0.05%, then 100 μL of an HRP-conjugated anti-biotin antibody (32 ng/mL) were added for 1 h at 37 °C. After washing, 100 μL of TMB were added and incubated for 20 min at RT in the dark. Finally, the absorbance was measured at 620 nm with a spectrophotometer (SpectroStar Nano, BMG Labtech). The data were analyzed with GraphPad using the statistical ANOVA test between samples.

Polysialylation assays were also performed at 27 °C for 4 h in 100 mM cacodylate buffer with 100 μM CMP-Sia (*i.e.* CMP-Neu5Ac, CMP-Neu5Gc or CMP-Kdn), 4 µg of the CD166/ALCAM and 230 μL of enzymatic source in a total volume of 1 mL. The enzymatic source with empty vector p3 × FLAG-CMV9 (Mock) and recombinant human ST8Sia IV were used as controls. Samples were cooled on ice, dialyzed overnight on 10 kDa dialysis membrane in ammonium bicarbonate buffer 50 mM and lyophilized. Samples were resuspended in 18 µL of RIPA buffer (Tris HCl 10 mM; NaCl 150 mM; Triton X-100 1%; pH 6.4) and treated or not with endoN (6.7 µg/mL) 1 h, 37 °C. CD166/ALCAM polysialylation was visualized by WB. Total samples were boiled for 5 min at 60 °C and resolved on a 6% SDS-PAGE as described previously.

### Chain length analysis of polySia by HPLC

Fish ST8Sia IV was used to polysialylate CD166/ALCAM as described in chapter Chemo-enzymatic synthesis of activated CMP-sialic acids and sialylation assays. The polysialylated fraction was isolated using affinity precipitation. Therefore, inactive endoN was coupled to tosyl-activated magnetic Dynabeads M-280 (Invitrogen, Carlsbad, CA) according to the manufacturer’s instructions as described previously^17,45^. Inactive endoN binds to polySia, but is not able to degrade the polymer. The samples were dialyzed against TBS for 2 h using Spectra/Por^®^ Biotech CE Tubing (MWCO: 50 kDa; Repligen, Rancho Dominguez, CA). Subsequently, the dialyzed samples were incubated with the beads for 30 min. After washing, polySia was eluted using 100 mM triethylamine, 150 mM NaCl and dried in a vacuum concentrator. To control the affinity precipitation, 10% of the eluate was analyzed by WB against polySia. The remaining 90% of the samples were used for HPLC analysis.

The polySia chain length determination of polysialylated CD166/ALCAM was performed using the 1,2-diamino-4,5-methylene-dioxybenzene (DMB)-HPLC strategy^46,47^. Briefly, the isolated polySia-CD166/ALCAM samples were dissolved in 80 µL DMB reagent (9 mM sodium hydrosulfite, 0.5 M mercaptoethanol, 20 mM trifluoroacetic acid (TFA), 0.61 mg/ml DMB) and incubated at 11 °C overnight. The reaction was stopped with 20 µL of 1 M NaOH for 1 h. The fluorescently labeled polySia chains were separated by anion exchange chromatography using a DNAPac PA-100 column (4 × 250 mm; 13 µm; Dionex). The eluents, MilliQ water (E1) and 2 M ammonium acetate (E2) were used with a flow rate of 1 mL/min following the gradient: 0 min = 0% E2, 5 min = 0% E2, 15 min = 13% E2, 30 min = 21% E2, 55 min = 33% E2, 100 min = 43% E2, 101 min = 100% E2, 110 min = 100% E2, 111 min = 0% E2, 145 min = 0% E2. Fluorescent signals were detected using an extinction wavelength of 372 nm and an emission wavelength of 456 nm.

### Quantitative analysis of polySia composition by HPLC

To quantitatively analyze the transfer of different CMP-Sia residues onto CD166/ALCAM by *Cma*ST8Sia IV, we established an endoN approach using centrifugal filter units. The analytical strategy is based on Ref.^48^. Microcon^®^ centrifugal filter units (MRCPRT010, Merck Millipore Ltd.; NMWL: 10 kDa) were prepared according to the manufacturer’s instructions. Polysialylation reaction was performed overnight at 37 °C in cacodylate buffer containing 100 µl fish ST8Sia IV culture medium, 150 ng CD166/ALCAM and 100 µM CMP-Sia (CMP-Neu5Ac or CMP-Neu5Gc or CMP-Kdn) in a total volume of 250 µl. To remove remaining free CMP-Sia, the samples were centrifuged with 14,000 × *g* at 4 °C until almost all the liquid was filtered. The samples were subsequently washed 5 more times with 250 µl 50 mM NH_4_HCO_3_ followed by centrifugal-filtration step. Thereafter, the volume was adjusted to 110 µl with 50 mM NH_4_HCO_3_ buffer. EndoN was added (1.34 µg/ml) to degrade polySia. In parallel, untreated samples (without endoN) of each CMP-Sia preparation were used as negative controls. Aliquots (10 µl) of all samples were collected for WB after 1 h at 37 °C. The remaining samples were centrifuged as described above and washed three more times. For quantitative Sia analysis, the complete flow-through (containing degraded sialic acid chains) was dried in a vacuum concentrator and dissolved in 0.2 N TFA. Hydrolysis was performed at 80 °C for 4 h. The hydrolyzed samples were dried and dissolved in 80 µl DMB reagent. After 2 h at 55 °C, the reaction was stopped with 20 µl 0.2 N NaOH. Resulting fluorescently labeled sialic acid residues were injected into a HPLC system (Nexera, Shimadzu) and separated using a Superspher^®^ 100 RP-18 end-capped column (250 mm × 40 mm, Merck-Hitachi, Darmstadt, Germany). A gradient with the eluents 92% MilliQ water, 4% ACN, 4% methanol, 0,1% TFA (E1) and 10% MilliQ water, 45% acetonitrile (ACN), 45% methanol, 0,1% TFA (E2) was applied with a flowrate of 0.25 ml/min as follows: 0 min = 0% E2, 2 min = 0% E2, 25 min = 2% E2, 35 min = 5% E2, 40 min = 50% E2, 45 min = 100% E2, 50 min = 100% E2, 51 min = 0% E2, 60 min = 0% E2. Fluorescent signals were detected using an extinction wavelength of 372 nm and an emission wavelength of 456 nm. Sialic acid standards were used to obtain a calibration line for quantification.

### Statistical analyses

The statistical analyses were performed with GraphPad Prism software (version 9.5.1) using One-way ANOVA and a multiple comparison Tukey test. Following labels are used: p < 0.05 (*); p < 0.01 (**); p < 0.001 (***); p < 0.0001 (***); ns, not significant (p ≥ 0.05).

### Ethics statements

These experiments had been approved by the Landesamt für Landwirtschaft, Lebensmittelsicherheit und Fischerei, Mecklenburg‐Vorpommern, according to the German animal welfare law (approval ID: LALLF M‐V/TSD/7221.3‐1‐069/18; November 2018) and all the methods were carried out in accordance with relevant Institutional guidelines and regulations and with the ARRIVE guidelines.

### Supplementary Information

Supplementary Information.

## Data Availability

Data and materials can be shared upon request to corresponding authors. The *C. maraena* transcriptome can be accessed at the NCBI Sequence Read Archive (Bioproject ID: PRJNA302355).
