# Supplementary material for: Salmonid polysialyltransferases to generate a variety of sialic acid polymers
Source: Sci Rep. 2023 Sep 20;13:15610. doi: 10.1038/s41598-023-42095-0 (PMC10511417; doi:10.1038/s41598-023-42095-0)
Supplement: Supplementary file 1 — Supplementary Information. [file 41598_2023_42095_MOESM1_ESM.pdf]

Supplementary Information

Salmonid polysialyltransferases to generate a variety of sialic acid polymers

Mathieu Decloquement[1], Marzia Tindara Venuto[2], Virginie Cogez[1], Anna Steinmetz[2], Céline Schulz[1], Cédric Lion[1], Maxence Noel[1], Vincent Rigolot[1], Roxana Elin Teppa[1], Christophe Biot[1], Alexander Rebl[3], Sebastian Peter Galuska[2*] and Anne Harduin-Lepers[1*]

[1]Univ. Lille, CNRS, UMR 8576 - UGSF - Unité de Glycobiologie Structurale et Fonctionnelle, F-59000 Lille, France
[2]Institute of Reproductive Biology, Leibniz Institute for Farm Animal Biology (FBN), Wilhelm-Stahl-Allee 2, 18196 Dummerstorf, Germany
[3]Institute of Genome Biology Leibniz Institute for Farm Animal Biology (FBN), Wilhelm-Stahl-Allee 2, 18196, Dummerstorf, Germany

*Corresponding author: Anne Harduin-Lepers, Unité de Glycobiologie Structurale et Fonctionnelle, UMR CNRS 8576, Université de Lille, Faculté des sciences et Technologies, 59655 Villeneuve d'Ascq, France. Phone: +33 320 33 62 46; Fax: +33 320 43 65 55; E-mail: anne.harduin-lepers@univ-lille.fr; orcid.org/0000-0002-1233-3799

*Corresponding author: Sebastian Peter Galuska, Research Institute of Reproductive Biology, (FBN), Wilhelm-Stahl-Allee 2, 18196 Dummerstorf, Germany. Phone: +49 38208 68-769; Fax: +49 38208 68-769 52; E-mail: Galuska.Sebastian@fbn-dummerstorf.de; orcid.org/0000-0001-5565-0252

Supplementary information

Supplementary Figure S1: Production in transfected HEK293 cells and biochemical characterization of recombinant polysialyltransferases *Cma* ST8Sia II-r1 and ST8Sia II-r2.

Supplementary Figure S2: SDS-PAGE and WB with the specific mAb735 to detect products formed after sialylation reaction with human and fish ST8Sia IV enzymes.

Supplementary Figure S3: ^{31}P NMR spectra of chemo-enzymatically synthesized activated CMP-Sias.

Supplementary Figure S4: Polysialylation status of HEK293 cells.

Supplementary Figure S5: Exosialylation of HEK293 cells with Mock enzyme source

Supplementary Figure S6: Exosialylation of HEK293 cells with *Cma*ST8Sia IV.

Supplementary Figure S7 Exosialylation of HEK293 cells with *Hsa*ST8Sia IV.

Supplementary Figure S8: On the top, amino acid sequence of the anti-polySia scFv of mAb735 and localization of hydrogen bound interactions.

Supplementary Figure S9: Original blots

Supplementary Table S-1: Description of sialylated glycanic structures of acceptor substrates used in MPSA.

Supplementary Table S-2: PCR oligonucleotides used for amplification of fish cDNAs

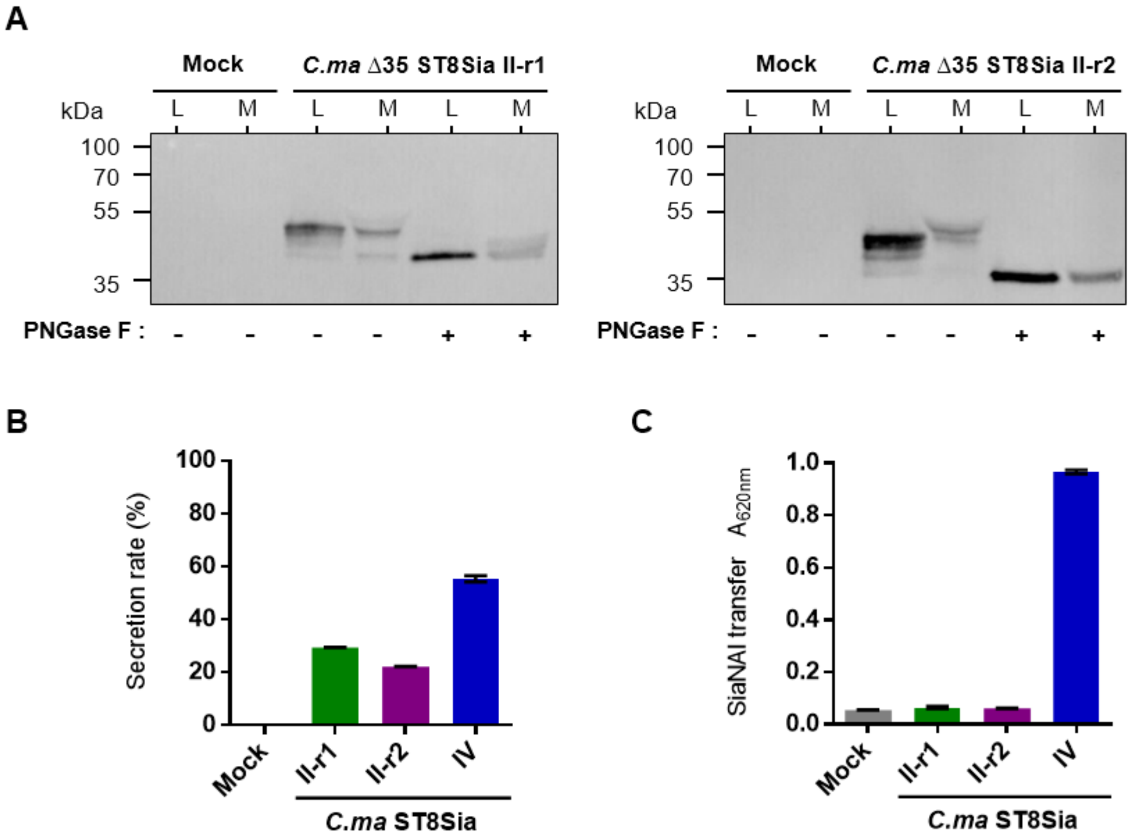

Figure S1: Production in transfected HEK293 cells and biochemical characterization on CD166/ALCAM using the MPSA of recombinant polysialyltransferases *Cma*ST8Sia II-r1 and ST8Sia II-r2. Plasmids for the expression of Δ28ST8Sia IV (blue), Δ35ST8Sia II-r1, (green) and Δ35ST8Sia II-r2 (purple) were transfected in HEK293 cells. Culture media and cells were collected 72 h post-transfection. Seven µg of cell lysates (L) and 20 µL of culture media (M) of HEK293 transfected cells with Δ35ST8Sia II-r1 (left panel) and Δ35ST8Sia II-r2 (right panel) were treated overnight with 50 U of PNGase F according to the manufacturer's instructions. Reaction products were denatured in Laemmli buffer and analyzed on WB. (**A**) Representative WB with PNGase-F treated and native samples. Samples were loaded, separated on SDS-PAGE (8 % acrylamide gel) and transferred on nitrocellulose membrane. WB was carried out with the anti-3×FLAG antibody (1 µg/mL). Original blot is presented in supplementary figure S9. Molecular weight markers are indicated on the left side. (**B**) Secretion efficiency of each *Cma* polysialyltransferases. WB signal intensities of culture media and cell lysate were analyzed by densitometry (ImageJ). Secretion of each isoform is reported in percentage (%) relative to total enzyme quantities in the culture media and cell lysates quantified on each WB (n=2). (**C**) Biochemical characterization on CD166/ALCAM using the MPSA. Sialylation reactions were performed in the MPSA 4 h at 27 °C using 400 ng of CD166/ALCAM, 100 µM of CMP-SiaNAl and 20 µL of either *Cma*Δ35ST8Sia II-r1 (green) or *Cma*Δ35ST8Sia II-r2 (purple) or 20 µL of *Cma*Δ28ST8Sia IV (blue). Mock transfected cell culture medium (grey) was used as negative control. Azido-PEG3-biotin was then covalently attached to the alkyne group of each transferred SiaNAl through copper catalyzed alkyne-azide cycloaddition (CuAAC) and biotin was detected by the anti-biotin antibody coupled with HRP (32 ng/mL). TMB (3,3′,5,5′-Tetramethylbenzidine) substrate was used and absorbance was measured with a microplate spectrophotometer at 620 nm. Error bars represent SEM (n=3).

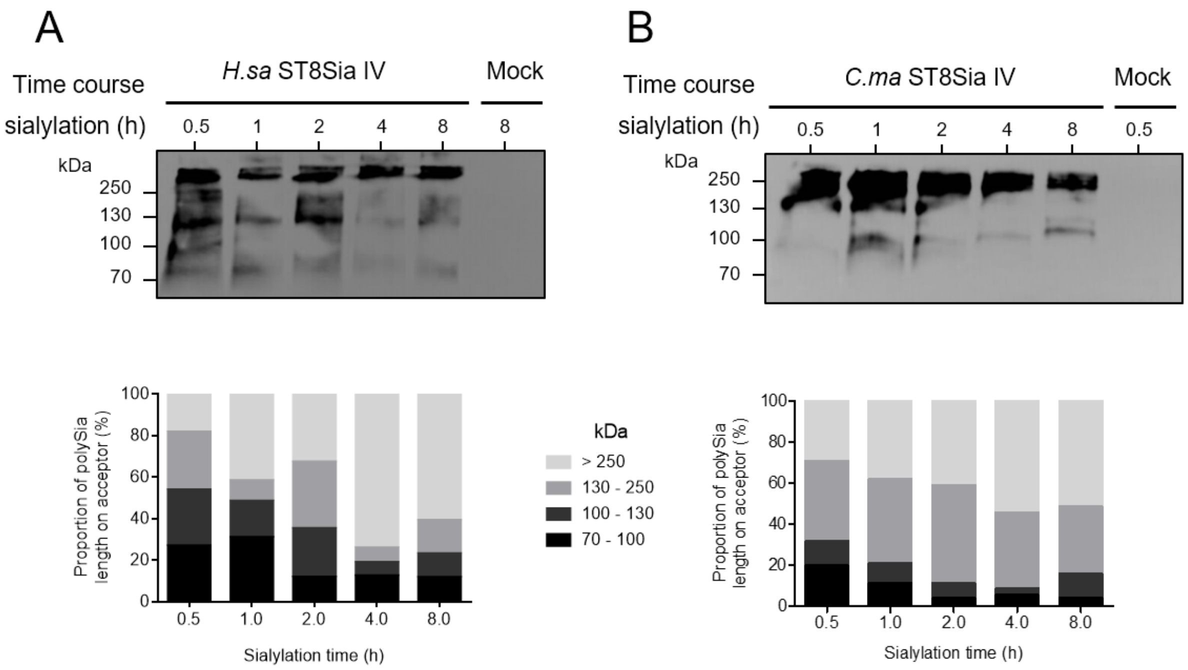

Figure S2: SDS-PAGE and Western blot with the specific mAb735 to detect products formed after sialylation reaction with human and fish ST8Sia IV enzymes. Time course sialylation reactions were performed from 0.5 to 8 h at 27 °C with 100 µM of natural donor substrate CMP-Neu5Ac on 6 µg of CD166/ALCAM with 150 ng of *H.sa* ST8Sia IV or 20 µL of *C.ma* ST8Sia IV or Mock. Sialylated products were heated for 5 min at 65 °C in Laemmli buffer to preserve the polySia chains, separated on SDS-PAGE (6 % acrylamide gel) and sialylated proteins were transferred onto nitrocellulose. Western blot analyses shown on the top were carried out with the mAb735. Original blots are presented in supplementary figure S9. Molecular weight markers are indicated on the left side. Densitometry analyses were performed with ImageJ and the percentage of polySia chains length according to band sizes is reported below for **A**) the human and **B**) the coregone enzyme (n=2).

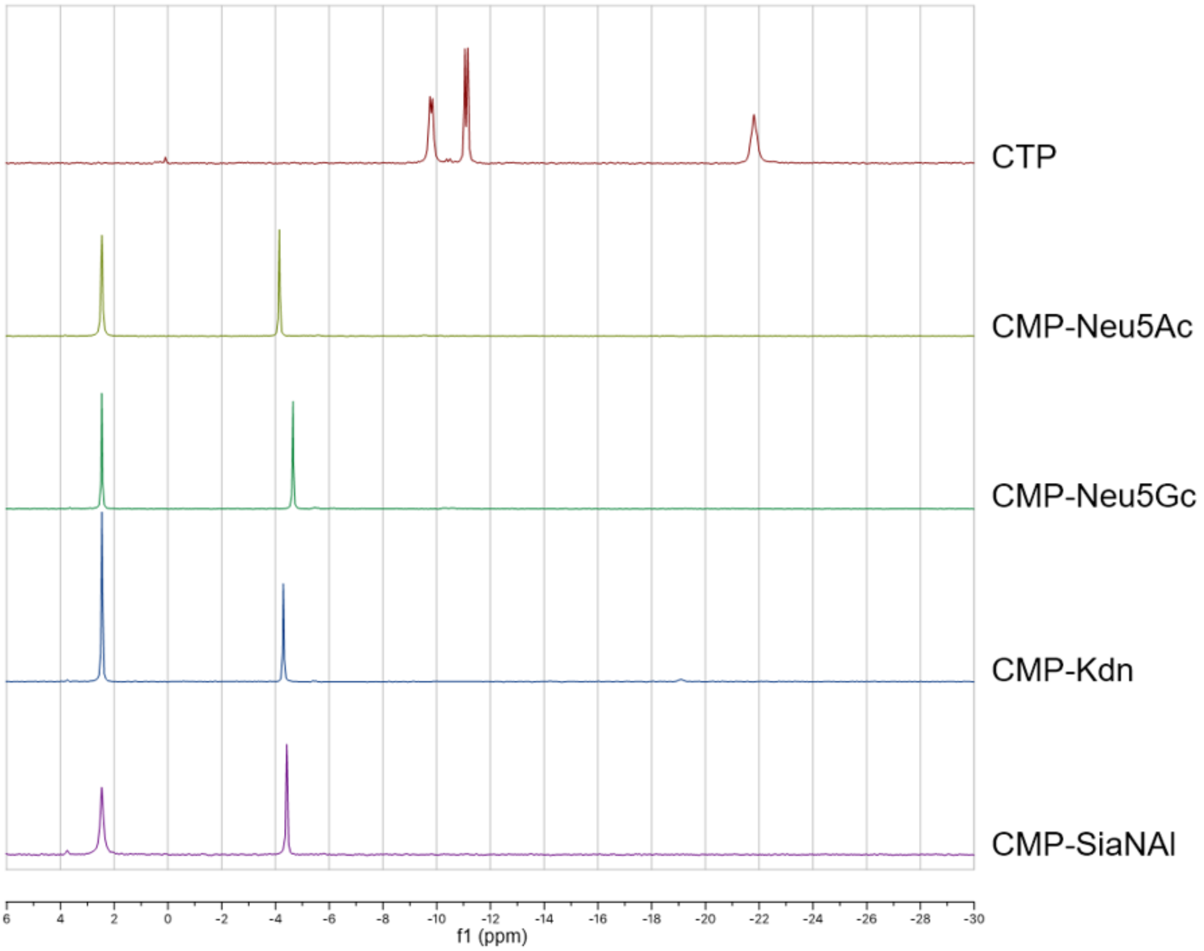

Figure S3: ^{31}P NMR spectra of chemo-enzymatically synthesized activated CMP-sialic acids. Enzymatic reactions were performed in equimolarity of CTP and each Sia (1:1) with 0.3 U CMP-Sialic acid Synthase (CSS) from *N. meningitidis* for CMP-SiaNAl, CMP-Neu5Ac and CMP-Neu5Gc and from rainbow trout for CMP-Kdn and 0.5 U PPase in Tris-HCl 100 mM, $MgCl_2$ 20 mM buffer (pH 8.5) during 1 h at 37 °C. Products integrity was checked by 1D NMR spectrometry of ^{31}P in a Brüker Avance II 400 MHz NMR spectrometer. Peaks shift (ppm) from CTP spectrum (red) after one hour of synthesis to CMP-Neu5Ac (olive), CMP-Neu5Gc (green), CMP-Kdn (blue) and CMP-SiaNAl (purple) spectra indicates 100 % formation and purity of the four products formed.

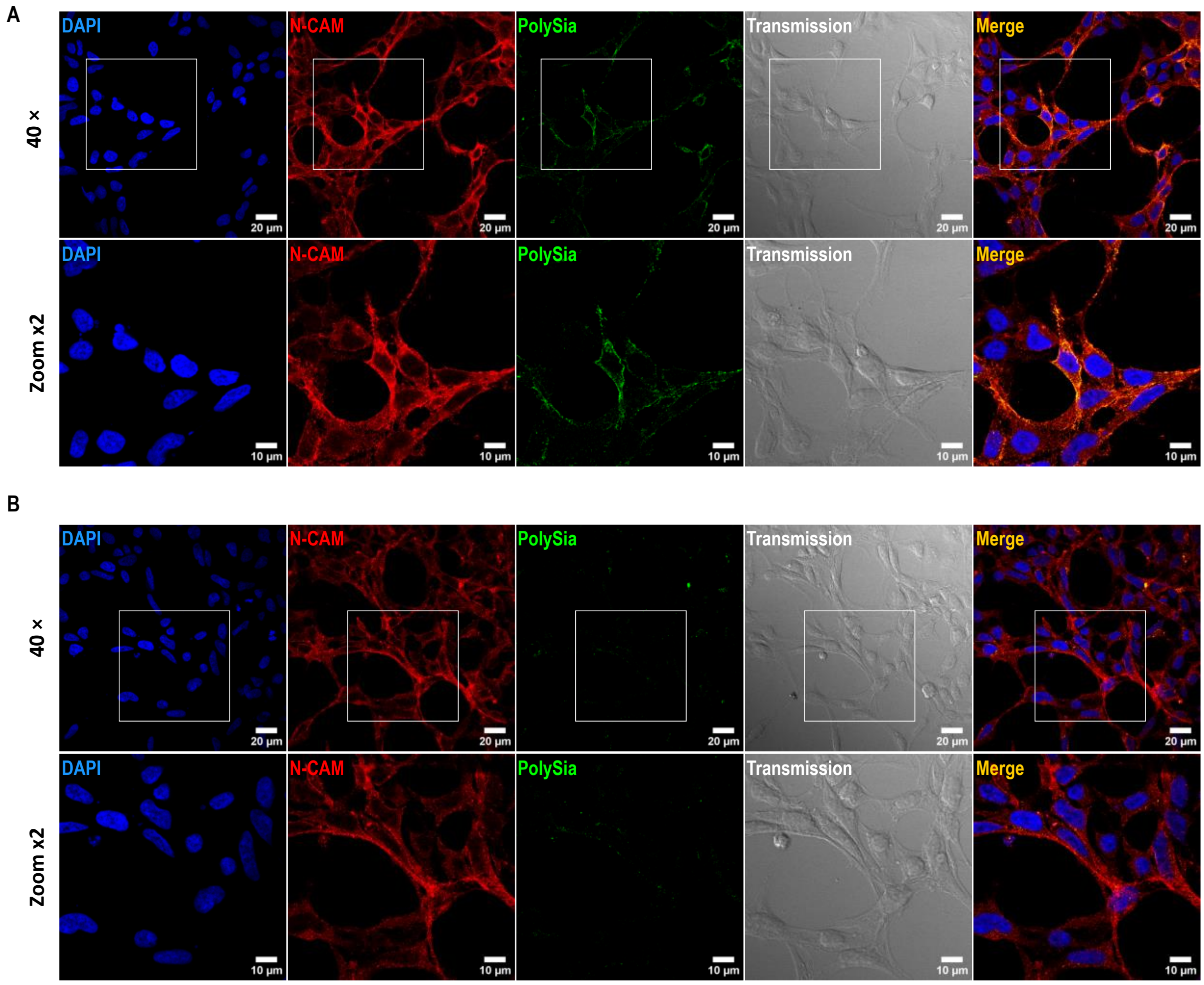

Figure S4: Polysialylation status of HEK293 cells. A) HEK293 cells without endoN treatment show polysialylation labelling on cell surface using mAb735 and anti-CD56/NCAM antibodies. B) After EndoN treatment, mAb735 detection signal (green) is abolished. Nuclei are stained with DAPI (blue). Outline of the cells is visualized by transmission in grey. DAPI, NCAM and polySia signals are superposed in Merge. Fluorescence was detected through a Carl Zeiss confocal microscope and Transmission correspond to transmitted light microscopy images. Scale bars: 20 µm for acquisitions at 40 × and 10 µm for the Zoom × 2 pictures.

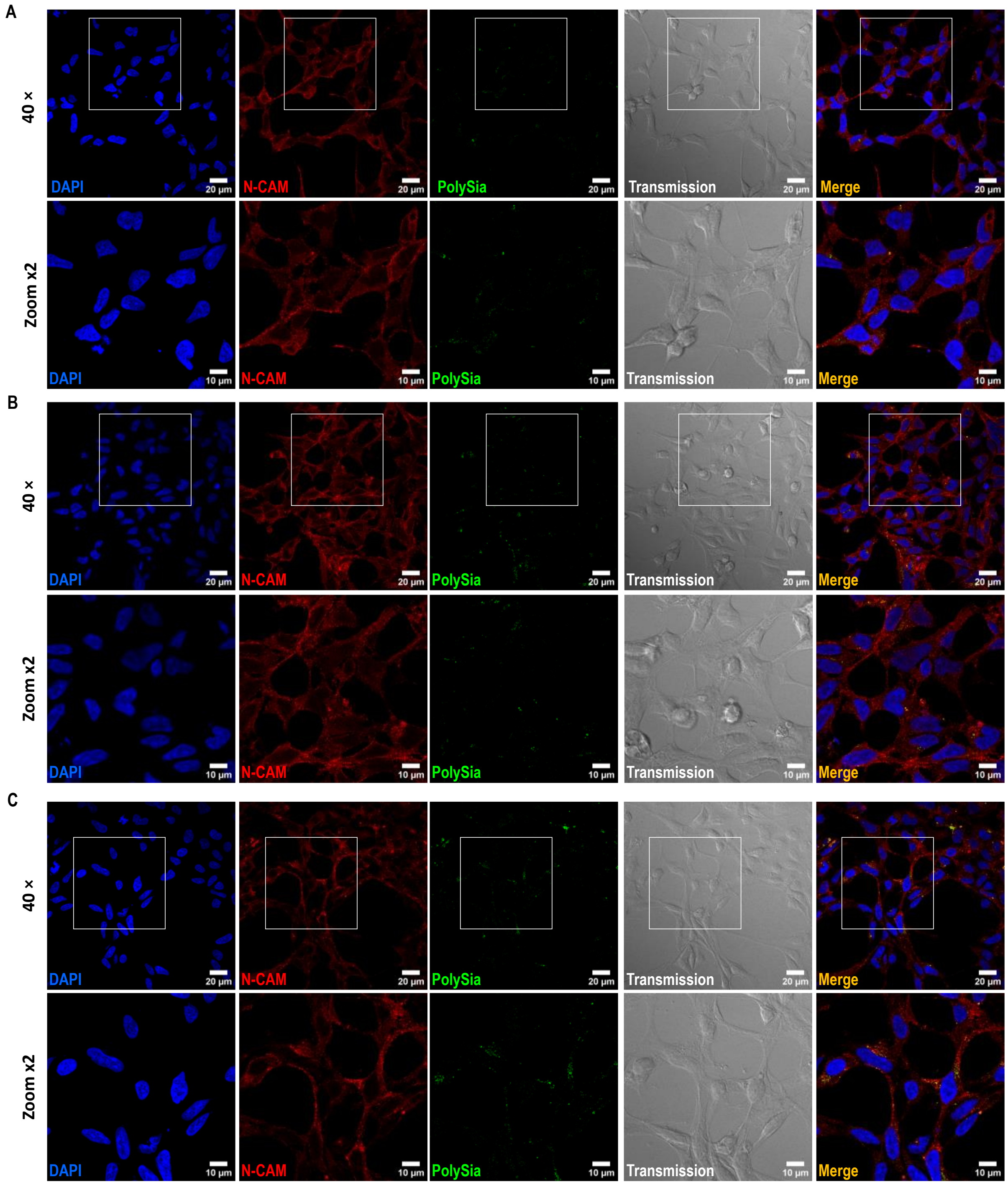

Figure S5: Exosialylation of HEK293 cells with Mock enzyme source: After EndoN treatment, sialylation reactions were carried out 4 h at 27°C on fixed cells with 40 µL of Mock medium and 500 µM of donor substrates: A) CMP-Neu5Ac, B) CMP-Neu5Gc and C) CMP-Kdn. Polysialylation on cell surface is detected using mAb735 antibody (green) and NCAM is detected using anti-CD56/NCAM (red) antibody. Nuclei are stained with DAPI (blue). Outline of the cells is visualized by transmission in grey. DAPI, NCAM and polySia signals are superposed in Merge. Fluorescence was detected through a Carl Zeiss confocal microscope and Transmission correspond to transmitted light microscopy images. Scale bars: 20 µm for acquisitions at 40 × and 10 µm for the Zoom × 2 pictures.

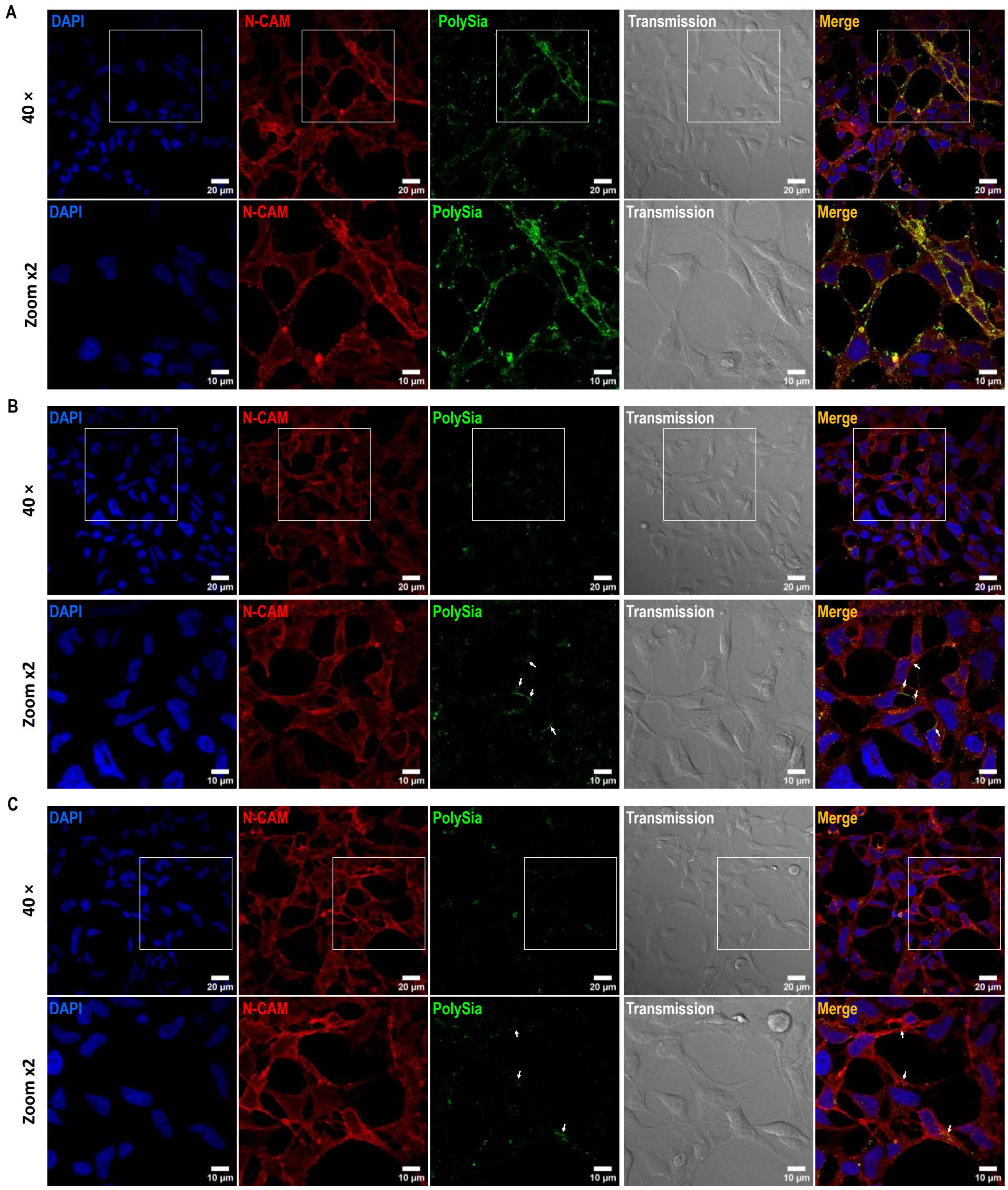

Figure S6: Exosialylation of HEK293 cells with *Cma*ST8Sia IV. After endoN treatment, sialylation reactions were carried out 4 h at 27 °C on fixed cells with 40 µL of *Cma* enzyme and 500 µM of donor substrates: A) CMP-Neu5Ac, B) CMP-Neu5Gc and C) CMP-Kdn. Polysialylation on cell surface is detected using mAb735 antibody and NCAM is detected using anti-CD56 antibody. Nuclei are stained with DAPI (blue). Outline of the cells is visualized by transmission in grey. DAPI, NCAM and polySia signals are superposed in Merge. Fluorescence was detected through a Carl Zeiss confocal microscope and Transmission correspond to transmitted light microscopy images. White arrows indicate polySias. Scale bars: 20 µm for acquisitions at 40 × and 10 µm for the Zoom × 2 pictures.

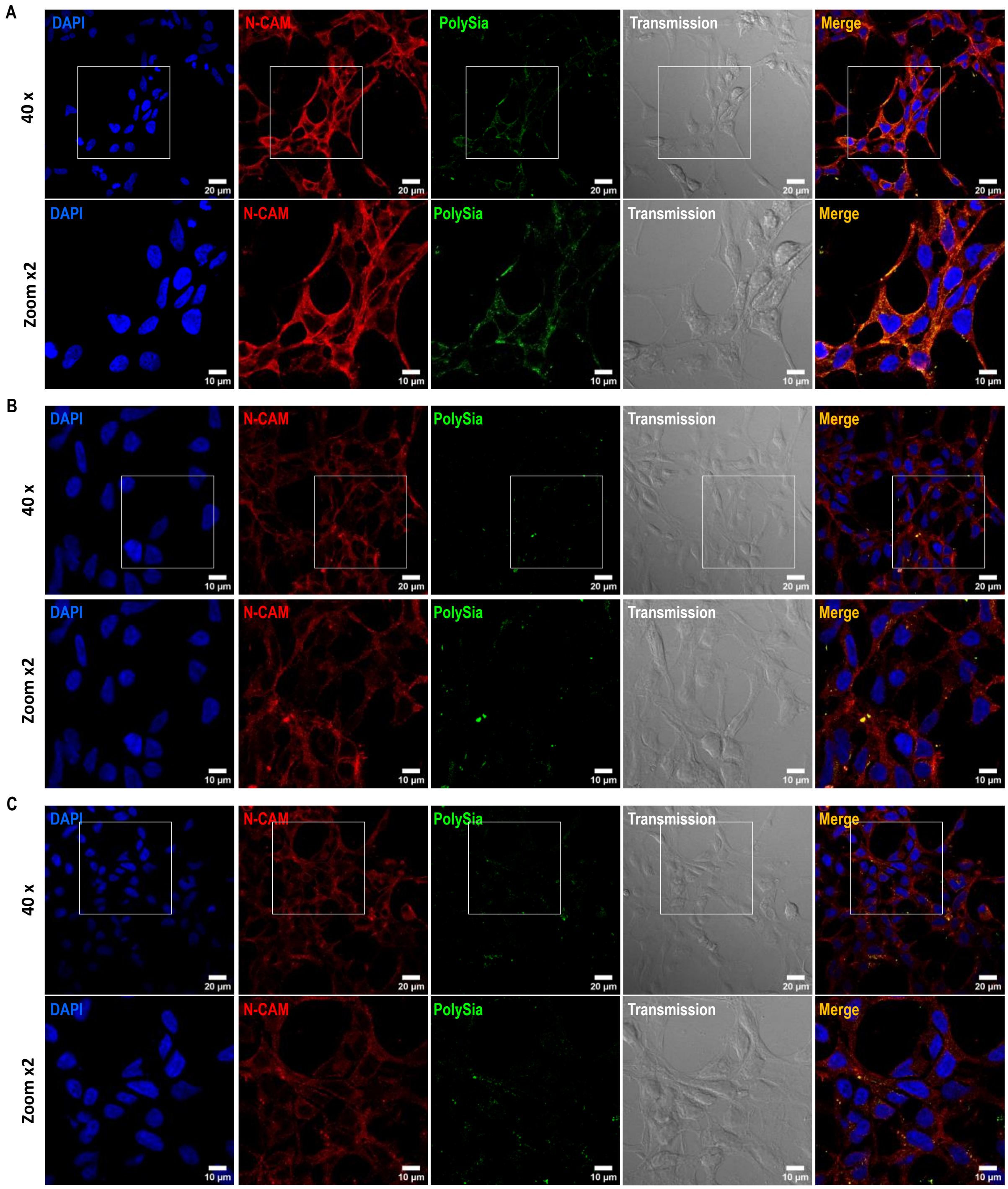

Figure S7 Exosialylation of HEK293 cells with *Hsa* ST8Sia IV. After endoN treatment, sialylation reactions were carried out 4 h at 27 °C on fixed cells with 200 ng of the human ST8Sia IV enzyme and 500 µM of donor substrates: A) CMP-Neu5Ac, B) CMP-Neu5Gc and C) CMP-Kdn. Polysialylation labelling on cell surface is detected using mAb735 and anti-CD56/NCAM antibodies. Nuclei are stained with DAPI (blue). Outline of the cells is visualized by transmission in grey. DAPI, NCAM and polySia signals are superposed in Merge. Fluorescence was detected through a Carl Zeiss confocal microscope and Transmission correspond to transmitted light microscopy images. Scale bars: 20 µm for acquisitions at 40 × and 10 µm for the Zoom ×2 pictures.

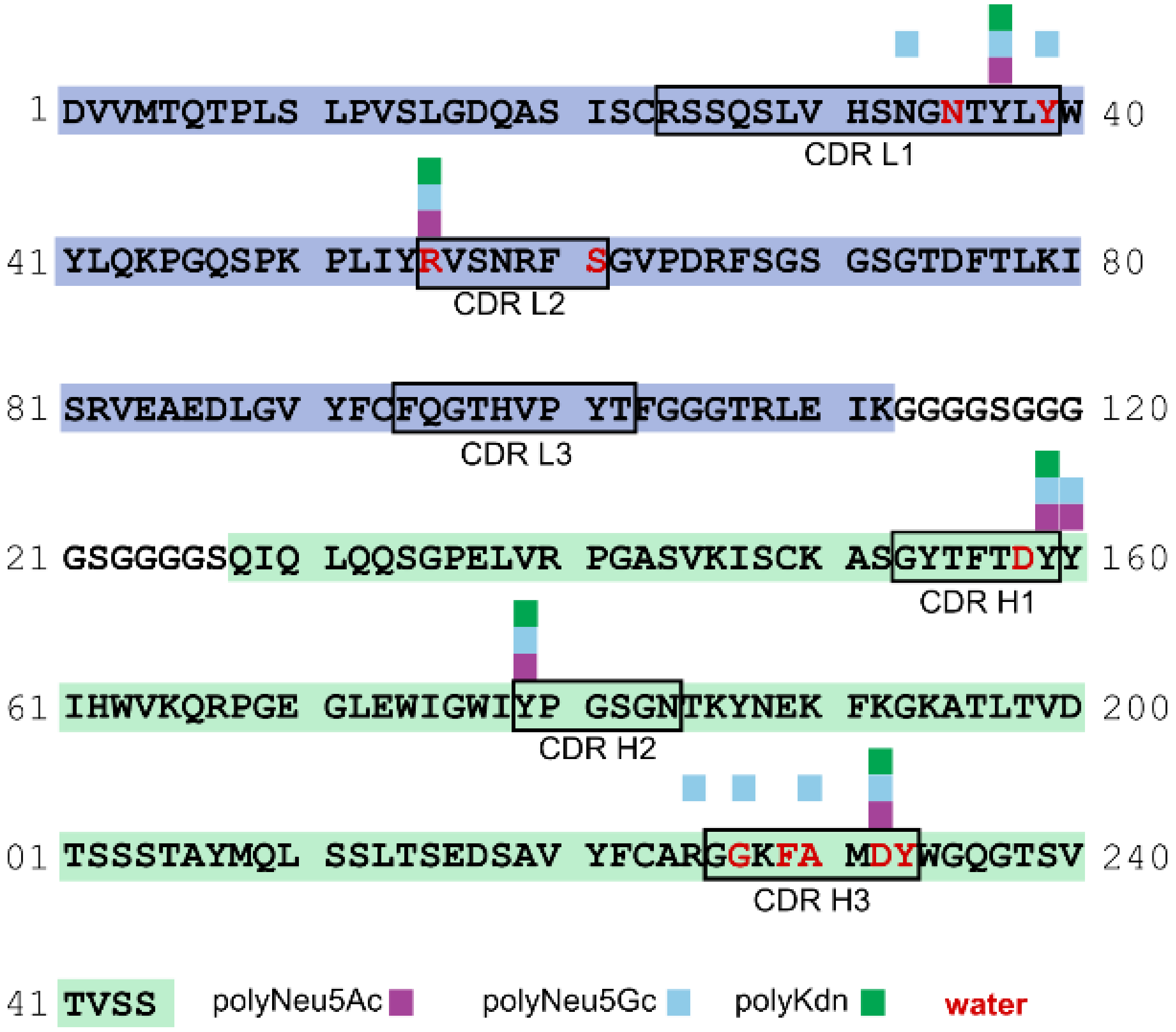

Amino Acid	CDR	Neu5Ac (equivalent)	Neu5Gc (equivalent)	Kdn (equivalent)
**Asn33**	L1		Sia3 (Sia7)	
**Asn35**	L1	Indirect	Indirect	Indirect
**Tyr37**	L1	Sia3 (Sia7)	Sia3 (Sia7)	Sia3 (Sia7)
**Tyr39**	L1	Indirect	Sia3 (Sia7)	Indirect

**Arg55**	L2	Sia2 (Sia6)<br>Indirect	Sia2 (Sia6)<br>Indirect	Sia2 (Sia6)<br>Indirect
**Ser61**	L2	Indirect	Indirect	Indirect
**Asp158**	H1	Indirect	Indirect	Indirect
**Tyr159**	H1	Sia2 (Sia6)	Sia2 (Sia6)	Sia2 (Sia6)
**Tyr160**	H1	Sia4 (Sia8)	Sia4 (Sia8)	
**Tyr179**	H2	Sia4 (Sia8)	Sia4 (Sia8)	Sia4 (Sia8)
**Arg225**			Sia2 (Sia6)	
**Gly227**	H3	Indirect	Sia2 (Sia6)	Indirect
**Phe229**	H3	Indirect	Indirect	Indirect
**Ala230**	H3	Indirect	Sia2 (Sia6)	Indirect
**Asp232**	H3	Sia2 (Sia6)<br>Indirect	Sia2 (Sia6)<br>Indirect	Sia2 (Sia6)<br>Indirect
**Tyr233**	H3	Indirect	Indirect	Indirect

Figure S8: On the top, amino acid sequence of the anti-polySia scFv of mAb 735 and localization of hydrogen bound interactions. VL and VH domains are colored in blue and green background, respectively. The complementarity-determining regions (CDRs) L1, L2, L3, H1, H2, H3 are indicated with black boxes. Colored boxes above the sequence mark residues that make direct interaction with polyNeu5Ac (purple), polyNeu5Gc (light blue) and polyKdn (green), whereas residues involved in water-mediated indirect interactions with the antigen are shown in red. Interactions are summarized in the table below.

Figure S9: Original and unprocessed images A. of figure 2; B. of supplemental figure S1; C. of figure 4; D. of figure 5; E. of supplemental figure S2; F. of figure 7

A. Figure 2: WB anti-FLAG *C.ma*ST8Sia IV

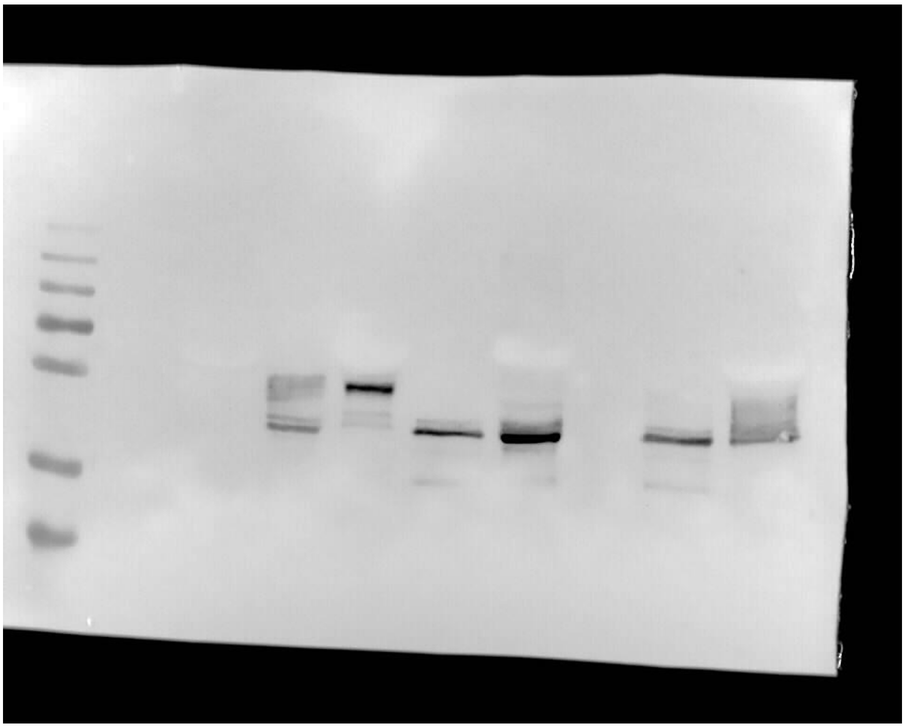

B. Supplemental figure S1: WB anti-Flag *C.ma*ST8Sia II-r1 and *C.ma*ST8Sia II-r2

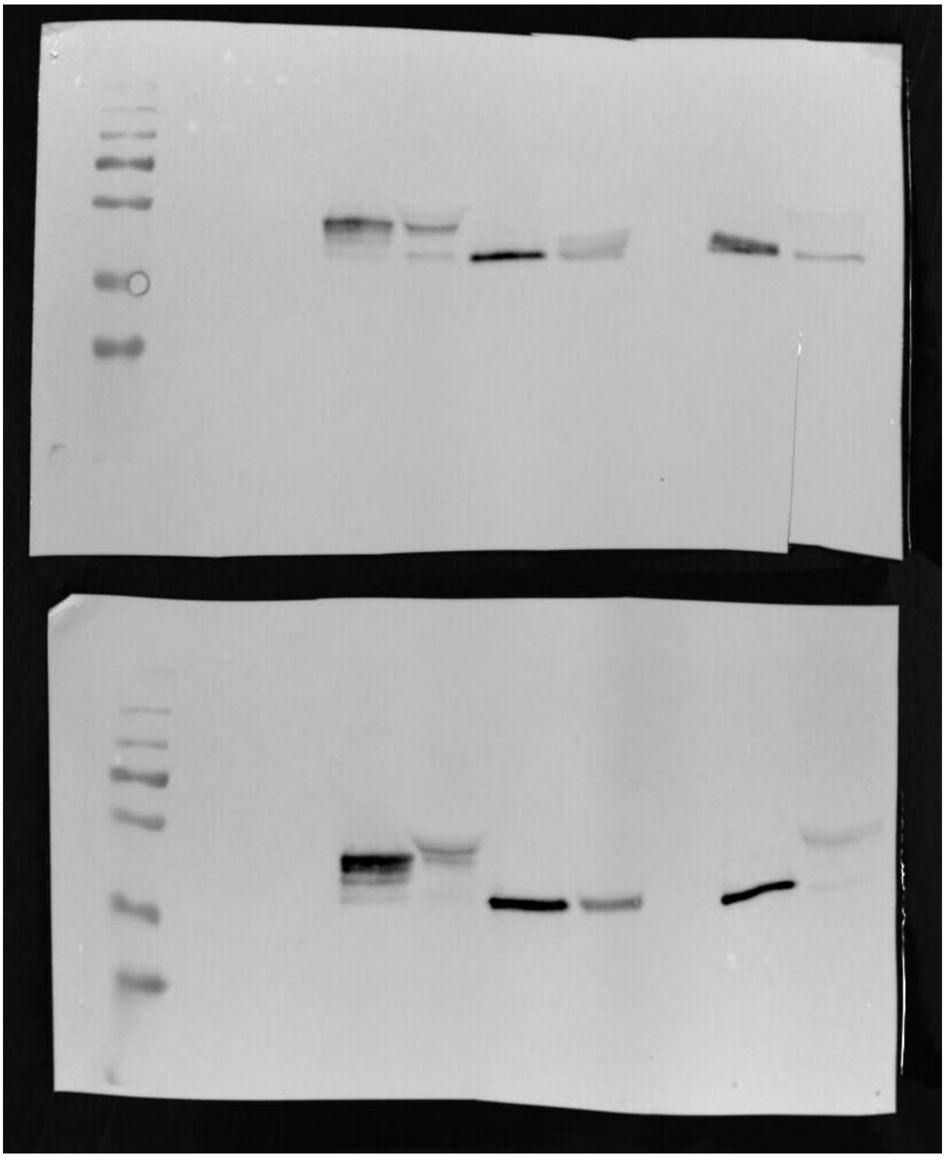

C. Figure 4: Controls by colorimetric analysis and WB with anti-polySia of the polysialylation status of the various actors of polysialylation prior sialylation reaction (Figure 4A).

Controls by WB with anti-polySia of the ALCAM polysialylation status after polysialylation reaction (Figure 4C)

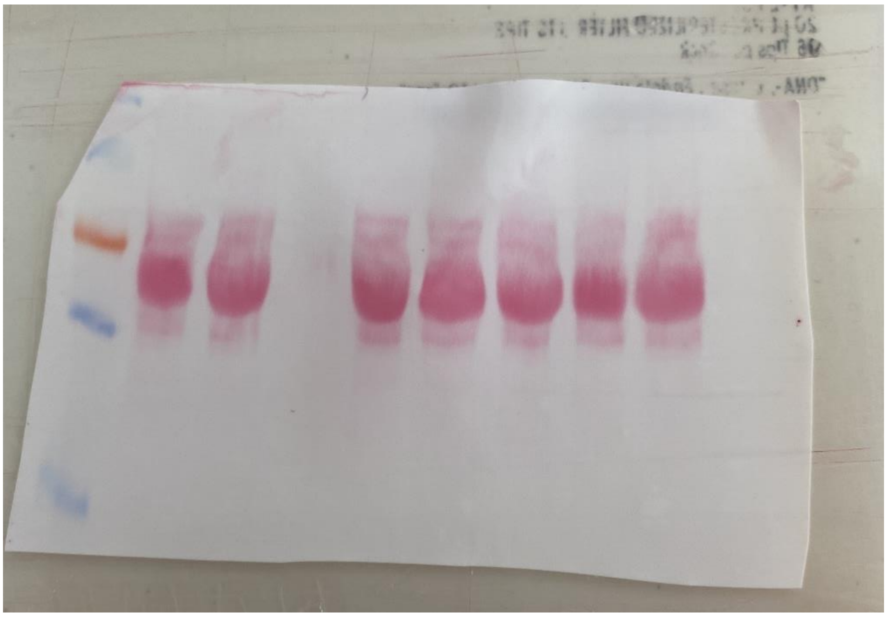

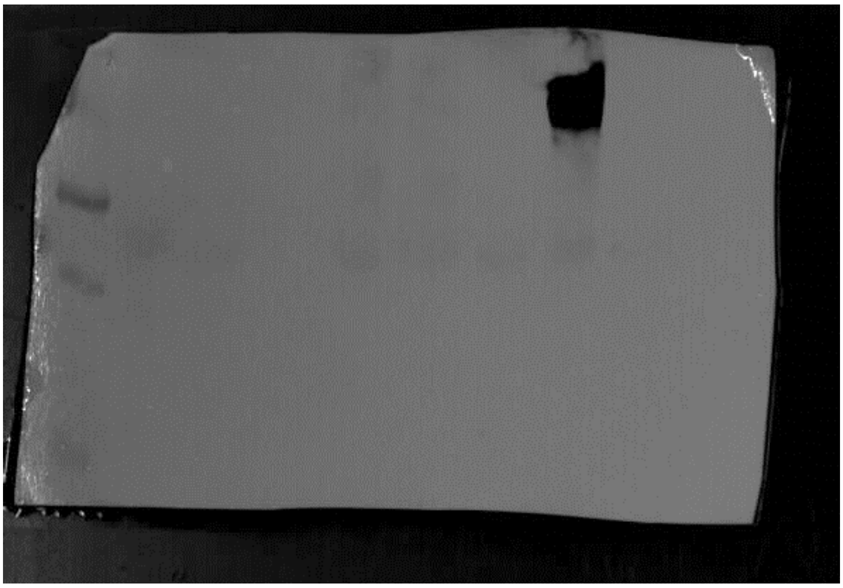

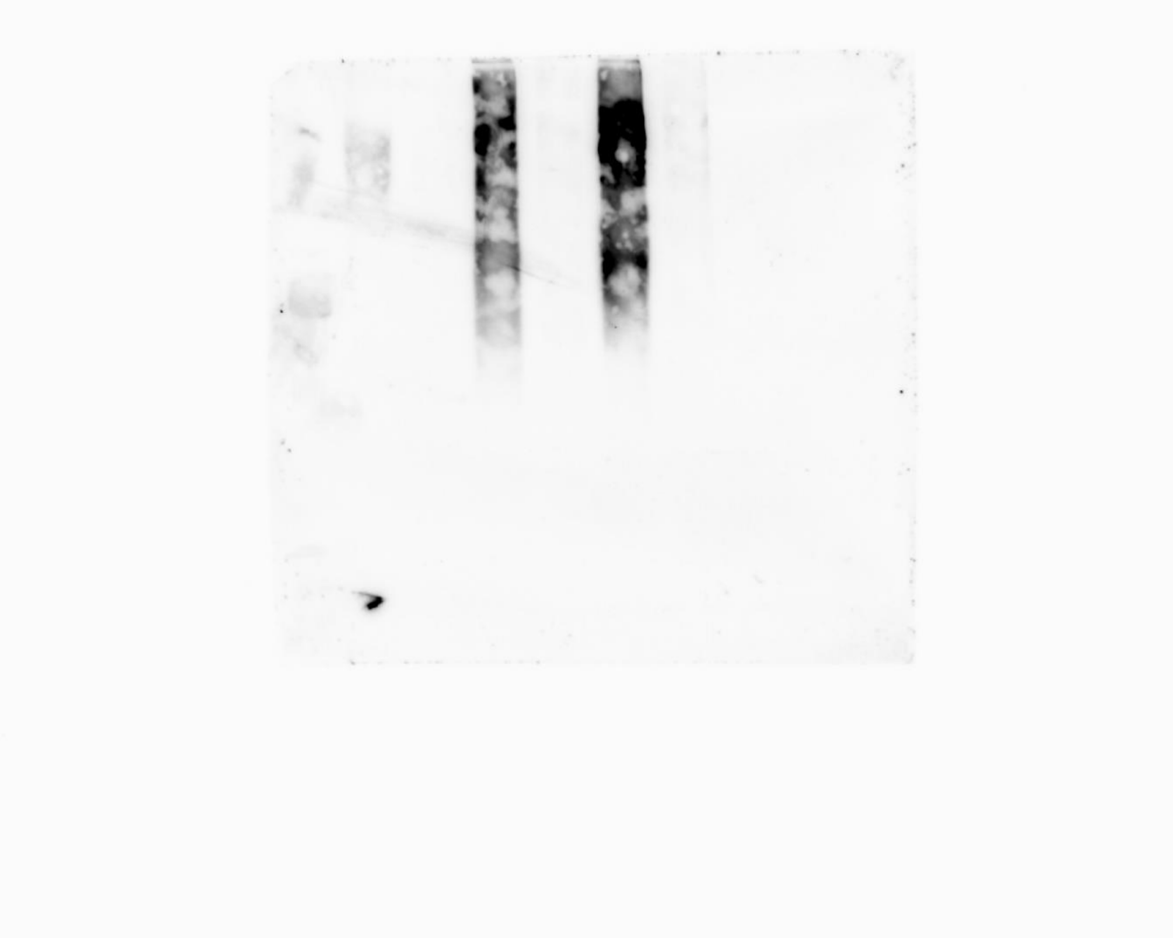

D. Figure 5 WB anti-polySia after sialylation with CMP-Neu5Ac, CMP-Neu5Gc and CMP-Kdn

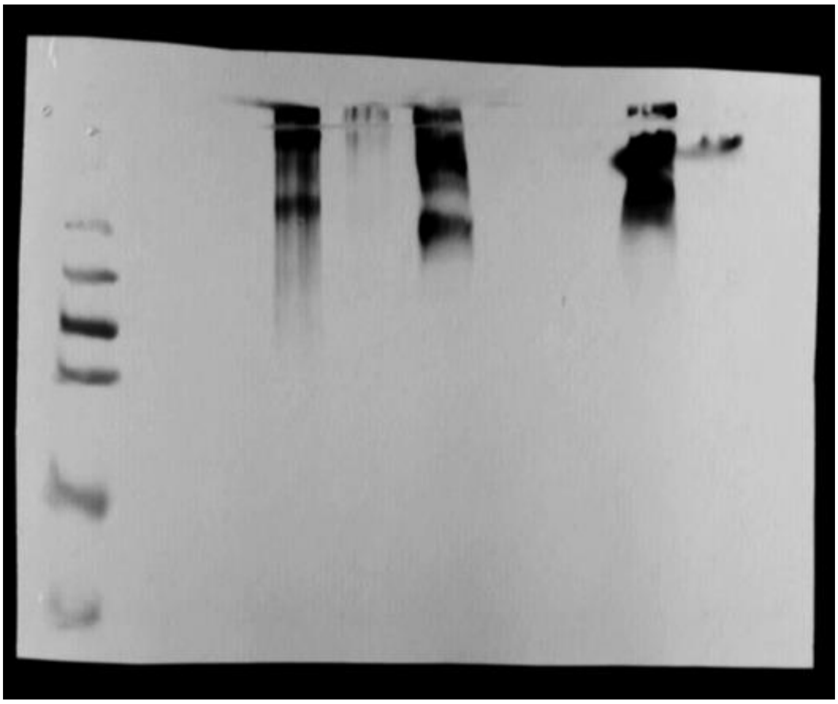

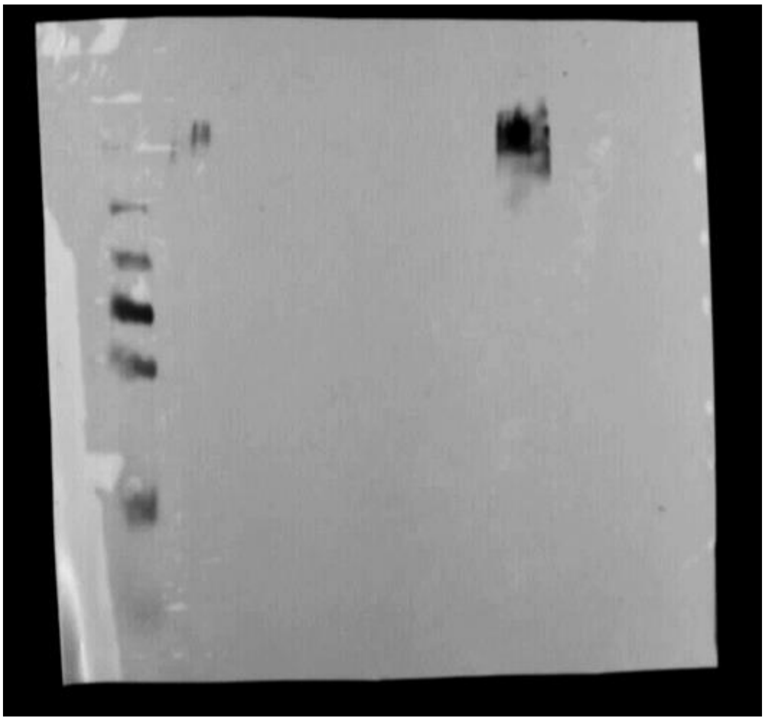

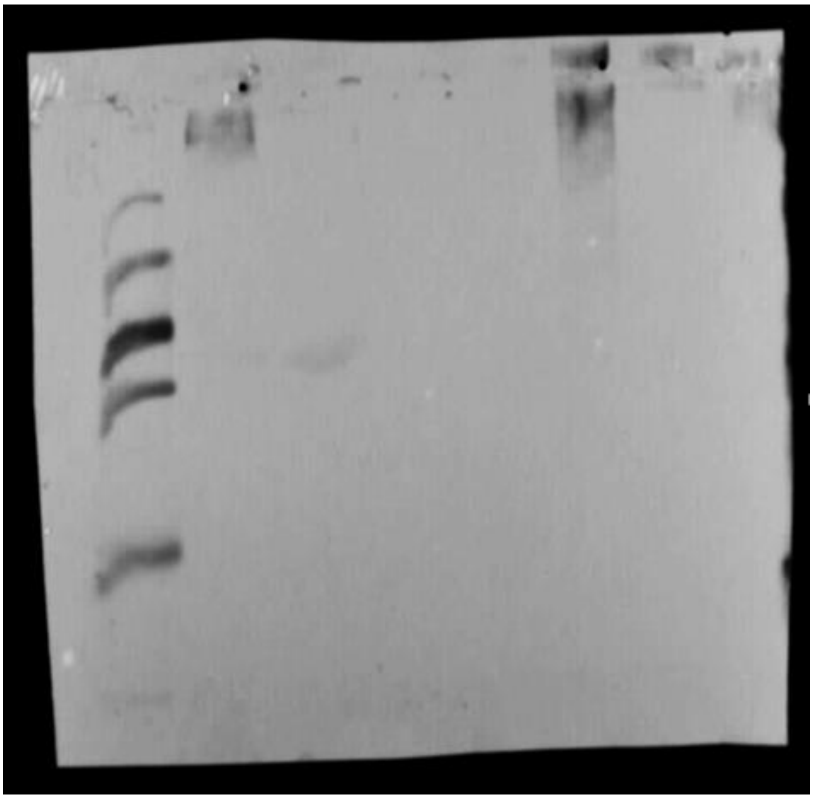

E. Supplemental figure S2: WB anti-polySia and time-course of polySia formation with CMP-Neu5Ac using human ST8Sia IV and fish ST8Sia IV

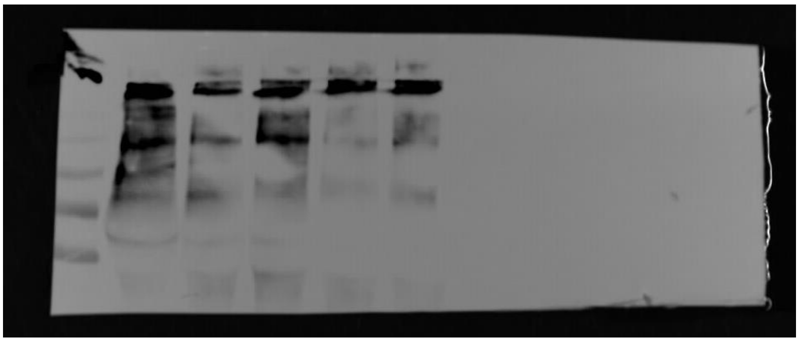

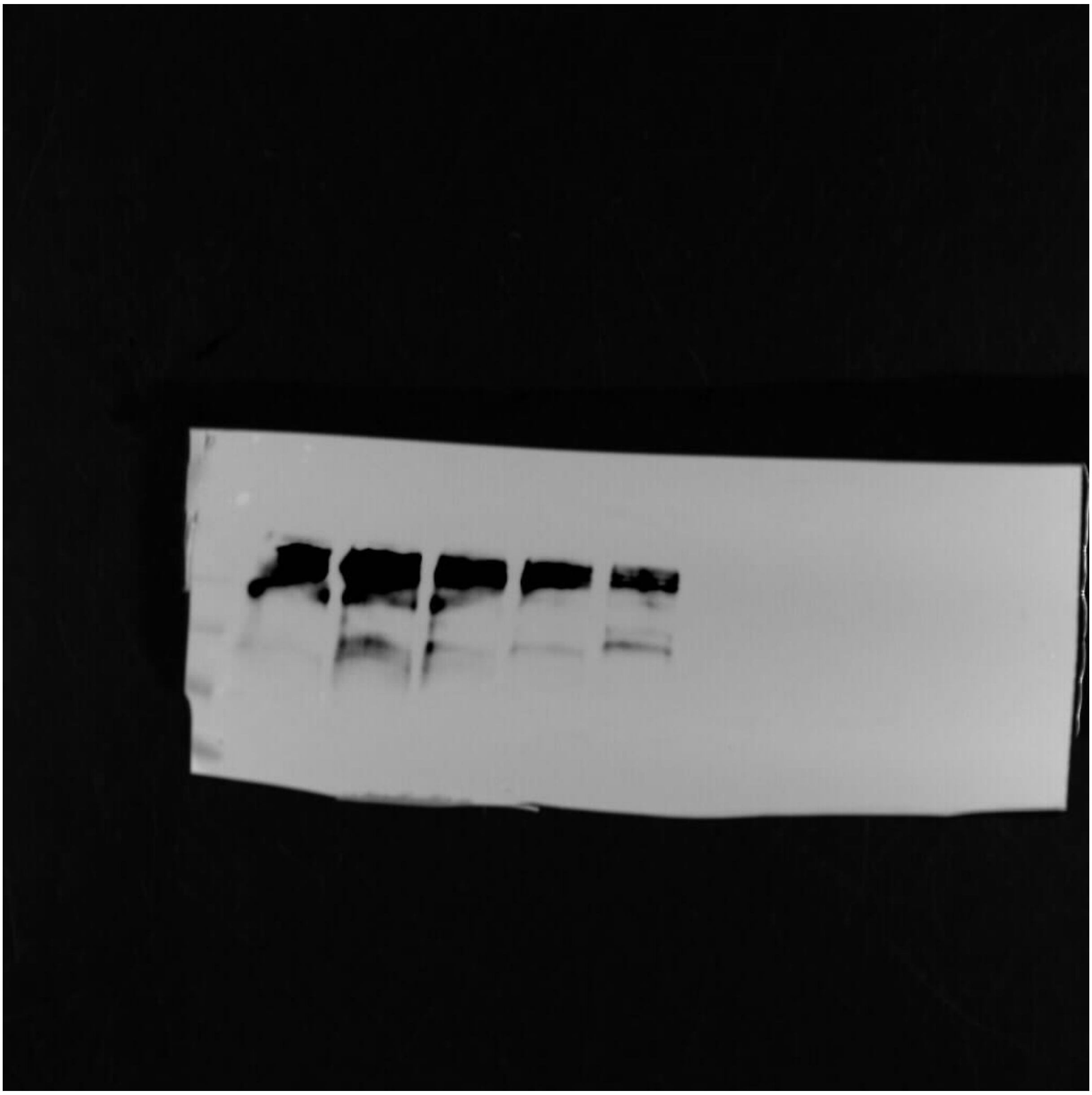

F. Figure 7: Chemiluminescence and colorimetric analysis of WB with anti-polySia

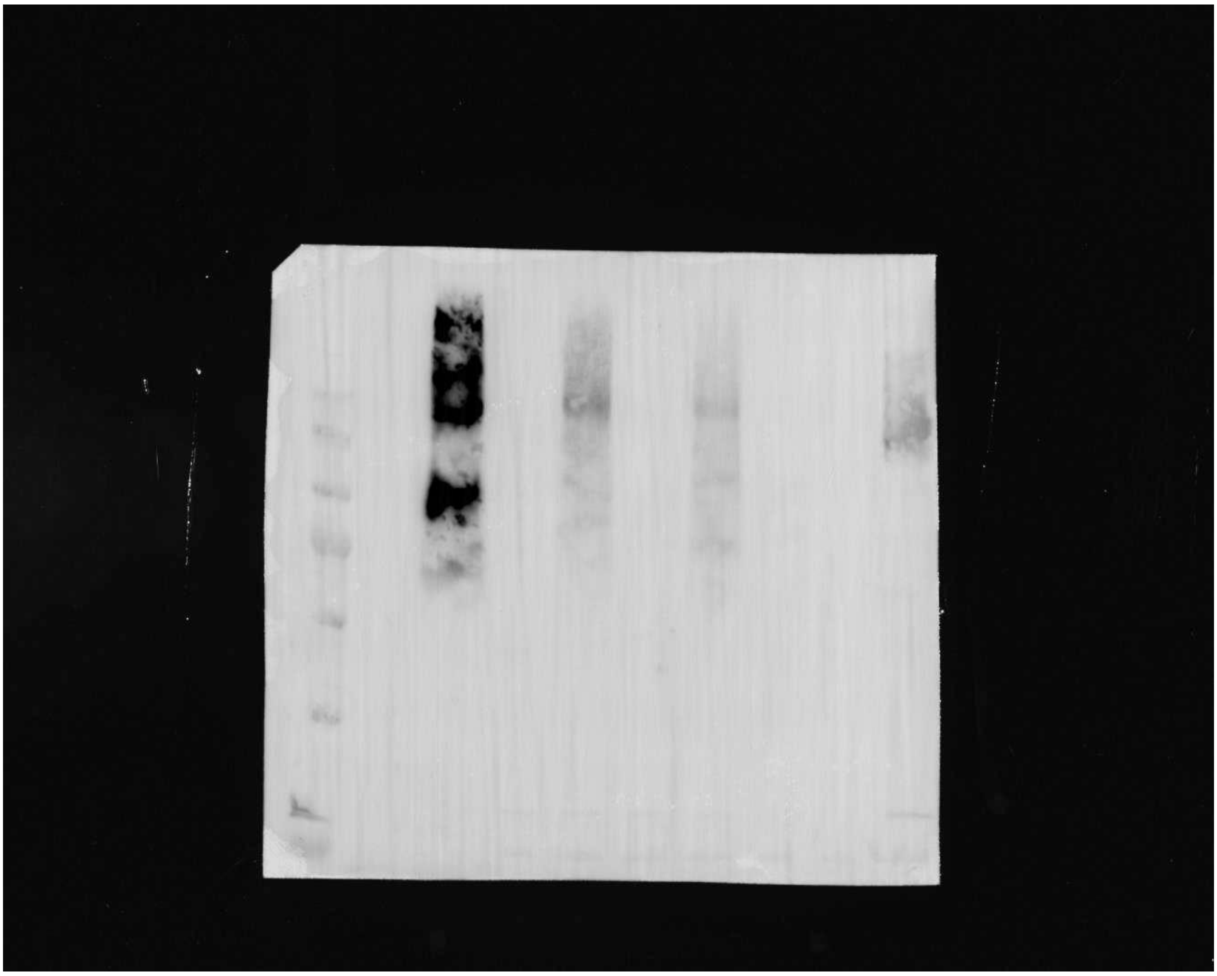

Table S-1: Description of sialylated glycanic structures of acceptor substrates used in enzymatic assay MPSA. Glycan representation was done with GlycoGlyph [1] according to the SNFG nomenclature [2].

Acceptor substrate	N-/O-glycans sialylated	Symbolic representation	Sialylated glycanic structure	References
Fetuin	N- : 3 tri-antennary sialylated N-glycans	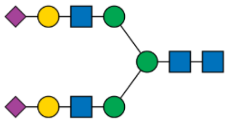	Neu5Acα2-6(3)Galβ1-4GlcNAcβ1-2Manα1-3[Neu5Acα2-6(3)Galβ1-4GlcNAcβ1-2Manα1-6]Manβ1-4GlcNAcβ1-4GlcNAc-Asn	[3]
		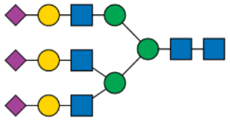	Neu5Acα2-6(3)Galβ1-4GlcNAcβ1-2[Neu5Acα2-6(3)Galβ1-4GlcNAcβ1-4]Manα1-3[Neu5Acα2-6(3)Galβ1-4GlcNAcβ1-2Manα1-6]Manβ1-4GlcNAcβ1-4GlcNAc-Asn	
		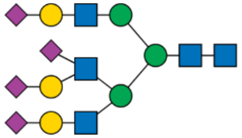	Neu5Acα2-6(3)Galβ1-4GlcNAcβ1-2[Neu5Acα2-6(3)Galβ1-4[Neu5Acα2-6]GlcNAcβ1-4]Manα1-3[Neu5Acα2-6(3)Galβ1-4GlcNAcβ1-2Manα1-6]Manβ1-4GlcNAcβ1-4GlcNAc-Asn	
	O- : core-1 mucin-type	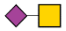	Neu5Acα2-6GalNAc-Ser/Thr	
		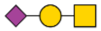	Neu5Acα2-3Galβ1-3GalNAc-Ser/Thr	

		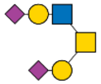	Neu5Acα2-3Galβ1-3[Neu5Acα2-3Galβ1-4GlcNAcβ1-6]GalNAc-Ser/Thr	
		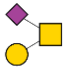	Galβ1-3[Neu5Acα2-6]GalNAc-Ser/Thr	
		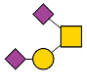	Neu5Acα2-3Galβ1-3[Neu5Acα2-6]GalNAc-Ser/Thr	
PSGP-L<br>PSGP-H	O- : core-1 and core-2 mucin-type disialylated (-L) or oligo- / poly-sialylated from 2 to 20 Sia (-H)	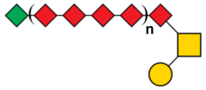	Kdnα2-8(Neu5Acylα2-8)$_{n\approx 2-20}$ Neu5Acylα2-6[Galβ1-3]GalNAc-Ser/Thr	4
		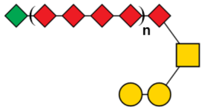	Kdnα2-8(Neu5Acylα2-8)$_{n\approx 2-20}$ Neu5Acylα2-6[Galβ1-4Galβ1-3]GalNAc-Ser/Thr	
		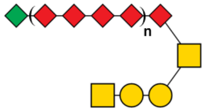	Kdnα2-8(Neu5Acylα2-8)$_{n\approx 2-20}$ Neu5Acylα2-6[GalNAcβ1-3Galβ1-4Galβ1-3]GalNAc-Ser/Thr	
		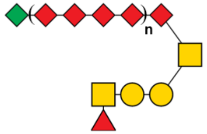	Kdnα2-8(Neu5Acylα2-8)$_{n\approx 2-20}$ Neu5Acylα2-6[Fucα1-3GalNAcβ1-3Galβ1-4Galβ1-3]GalNAc-Ser/Thr	
		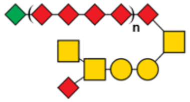	Kdnα2-8(Neu5Acylα2-8)$_{n\approx 2-20}$ Neu5Acylα2-6[GalNAcβ1-4[Neu5Acylα2-3]GalNAcβ1-3Galβ1-4Galβ1-3]GalNAc-Ser/Thr	

		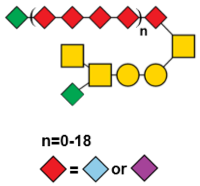	Kdnα2-8(Neu5Acylα2-8)$_{n\approx 2\text{-}20}$ Neu5Acylα2-6[GalNAcβ1-4[Kdnα2-3]GalNAcβ1-3Galβ1-4Galβ1-3]GalNAc-Ser/Thr	
Bovine Submaxillary Mucin (BSM)	O- : Core -1, Core -2, Core -3, Core -5	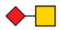	Neu5Acylα2-6GalNAc-Ser/Thr	5
		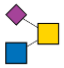	Neu5Acα2-6[GlcNAcβ1-3]GalNAc-Ser/Thr	
		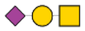	Neu5Acα2-3Galβ1-3GalNAc-Ser/Thr	
		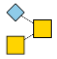	Neu5Gcα2-6[GalNAcα1-3]GalNAc-Ser/Thr	
		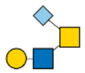	Neu5Gcα2-6[Galβ1-4GlcNAcβ1-3]GalNAc-Ser/Thr	
		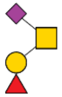	Neu5Acα2-6[Fucα1-3(4)Galβ1-3]GalNAc-Ser/Thr	
		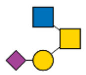	Neu5Acα2-3Galβ1-3[GlcNAcβ1-6]GalNAc-Ser/Thr	
		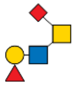	Neu5Acylα2-6[Fucα1-3(4)Galβ1-4GlcNAcβ1-3]GalNAc-Ser/Thr	

		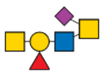	Neu5Acα2-6[GalNAcβ1-4[Fucα1-3]Galβ1-4GlcNAcβ1-3]GalNAc-Ser/Thr	
		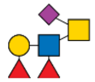	Neu5Acα2-6[Fucα1-3(4)Galβ1-4[Fucα1-3]GlcNAcβ1-3]GalNAc-Ser/Thr	
		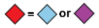		
Orosomucoid (α-1-acid glycoprotein)	N- ; di-, tri- and tetra-antennary LacNAc sialylated N-glycans	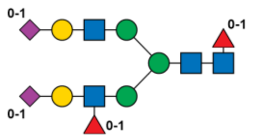	(Neu5Acα2-6(3))$_{0-1}$Galβ1-4[(Fucα1-3)$_{0-1}$]GlcNAcβ1-2Manα1-3[(Neu5Acα2-6(3))$_{0-1}$Galβ1-4GlcNAcβ1-2Manα1-6]Manβ1-4GlcNAcβ1-4[(Fucα1-6)$_{0-1}$]GlcNAc-Asn	6
		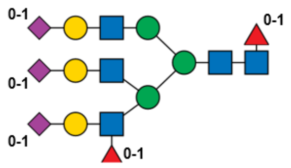	(Neu5Acα2-6(3))$_{0-1}$Galβ1-4[(Fucα1-3)$_{0-1}$]GlcNAcβ1-2[(Neu5Acα2-6(3))$_{0-1}$Galβ1-4GlcNAcβ1-4]Manα1-3[(Neu5Acα2-6(3))$_{0-1}$Galβ1-4GlcNAcβ1-2Manα1-6]Manβ1-4GlcNAcβ1-4[(Fucα1-6)$_{0-1}$]GlcNAc-Asn	
		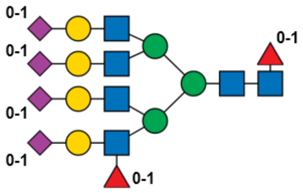	(Neu5Acα2-6(3))$_{0-1}$Galβ1-4[(Fucα1-3)$_{0-1}$]GlcNAcβ1-2[(Neu5Acα2-6(3))$_{0-1}$Galβ1-4GlcNAcβ1-4]Manα1-3[(Neu5Acα2-6(3))$_{0-1}$Galβ1-4GlcNAcβ1-2[(Neu5Acα2-6(3))$_{0-1}$Galβ1-4GlcNAcβ1-4]Manα1-6]Manβ1-4GlcNAcβ1-4[(Fucα1-6)$_{0-1}$]GlcNAc-Asn	
		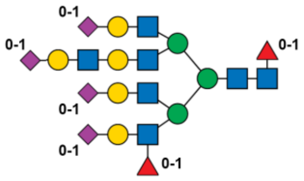	(Neu5Acα2-6(3))$_{0-1}$Galβ1-4[(Fucα1-3)$_{0-1}$]GlcNAcβ1-2[(Neu5Acα2-6(3))$_{0-1}$Galβ1-4GlcNAcβ1-4]Manα1-3[(Neu5Acα2-6(3))$_{0-1}$Galβ1-4GlcNAcβ1-4Galβ1-4GlcNAcβ1-2[(Neu5Acα2-6(3))$_{0-1}$Galβ1-4GlcNAcβ1-	

			4]Manα1-6]Manβ1-4GlcNAcβ1-4[(Fucα1-6)$_{0-1}$]GlcNAc-Asn	
		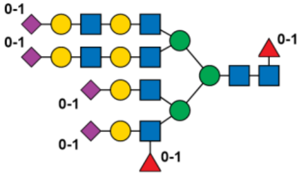	(Neu5Acα2-6(3))$_{0-1}$Galβ1-4[(Fucα1-3)$_{0-1}$]GlcNAcβ1-2[(Neu5Acα2-6(3))$_{0-1}$Galβ1-4GlcNAcβ1-4]Manα1-3[(Neu5Acα2-6(3))$_{0-1}$Galβ1-4GlcNAcβ1-4Galβ1-4GlcNAcβ1-2[(Neu5Acα2-6(3))$_{0-1}$Galβ1-4GlcNAcβ1-4Galβ1-4GlcNAcβ1-4]Manα1-6]Manβ1-4GlcNAcβ1-4[(Fucα1-6)$_{0-1}$]GlcNAc-Asn	
CD166/ALCAM	N- : bi-, tri- and tetra-antennary sialylated N-glycans (α2.6- and α2.3-Sia)	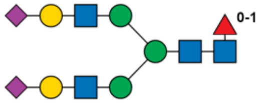	Neu5Acα2-6Galβ1-4GlcNAcβ1-2Manα1-3[Neu5Acα2-6Galβ1-4GlcNAcβ1-2Manα1-6]Manβ1-4GlcNAcβ1-4[(Fucα1-6)$_{0-1}$]GlcNAc-Asn	7, 8
		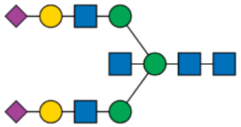	Neu5Acα2-6Galβ1-4GlcNAcβ1-2Manα1-3[GlcNAcβ1-4][Neu5Acα2-6Galβ1-4GlcNAcβ1-2Manα1-6]Manβ1-4GlcNAcβ1-4GlcNAc-Asn	
		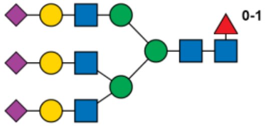	Neu5Acα2-3(6)Galβ1-4GlcNAcβ1-2[Neu5Acα2-3(6)Galβ1-4GlcNAcβ1-4]Manα1-3[Neu5Acα2-3(6)Galβ1-4GlcNAcβ1-2Manα1-6]Manβ1-4GlcNAcβ1-4[(Fucα1-6)$_{0-1}$]GlcNAc-Asn	

		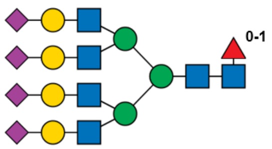	Neu5Acα2-3Galβ1-4GlcNAcβ1-2[Neu5Acα2-3Galβ1-4GlcNAcβ1-4]Manα1-3[Neu5Acα2-3Galβ1-4GlcNAcβ1-2[Neu5Acα2-3Galβ1-4GlcNAcβ1-2]Manα1-6]Manβ1-4GlcNAcβ1-4[(Fucα1-6)$_{0-1}$]GlcNAc-Asn	
DNase I	N- : LacNAc sialylated N-glycans	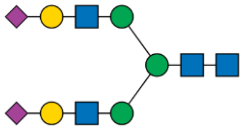	Neu5Acα2-6(3)Galβ1-4GlcNAcβ1-2Manα1-3[Neu5Acα2-6(3)Galβ1-4GlcNAcβ1-2Manα1-6]Manβ1-4GlcNAcβ1-4GlcNAc-Asn	9
NRP-2	O- : Core-1, Core-2	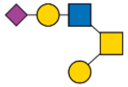	Neu5Acα2-3Galβ1-4GlcNAcβ1-6[Galβ1-3]GalNAc-Ser/Thr	10
		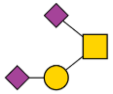	Neu5Acα2-3Galβ1-3[Neu5Acα2-6]GalNAc-Ser/Thr	
		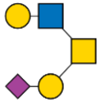	Galβ1-4GlcNAcβ1-6[Neu5Acα2-3Galβ1-3]GalNAc-Ser/Thr	
		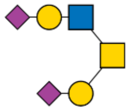	Neu5Acα2-3Galβ1-4GlcNAcβ1-6[Neu5Acα2-3Galβ1-3]GalNAc-Ser/Thr	

Table S-2: List of sense and antisense oligonucleotides used to generate cDNA encoding truncated form of the *C. maraena st8sia2 r-1*, *st8sia2 r-2* and *st8sia4* lacking their first 35, 35 or 28 amino acid residues, respectively. CDNAs were amplified by PCR using previous construct p3×FLAG-CMV10 as DNA templates for *st8sia4* and *st8sia2 r-1* and pcDNA3.1 for *st8sia2 r-2*, leading to Δ35 ST8Sia II-r1, Δ35 ST8Sia II-r2 or Δ28 ST8Sia IV constructs.

st8sia4:	Full-length	5'-CCC*AAGCTT*ATGCTCTCACGGAAACGC-3' (sense)
		5'-CGG*GGTACC*TTAAGATTCGCACTTCGAAGTCG-3' (antisense)
	Δ28ST8Sia IV	5'-GCA*AAGCTT*GAAGAACATCAGGAAGCTCAAGTC-3' (sense)
		5'-CGG*GGTACC*TTAAGATTCGCACTTCGAAGTCG-3' (antisense)
st8sia2 r-1:	Full-length	5'-CCC*AAGCTT*ATGCAGTTAGAATTCCGAACGCTG-3' (sense)
		5'-CGG*GGTACC*TTACATTCCTGCATCACAAGAGCC-3' (antisense)
	Δ35ST8Sia II-r1	5'-AAA*AAGCTT*GGAGGTTCCAGAACATTGTACTTG-3' (sense)
		5'-CTTGCCCCTTGCTCCATACCAC-3' (antisense)
st8sia2 r-2:	Full-length	5'-CCC*AAGCTT*ATGCAGTTAGAATTCCGAACATTG-3' (sense)
		5'-CGG*GGTACC*TTACGTTCCTGGATCACAAGAGCC-3' (antisense)
	Δ35ST8Sia II-r2	5'-AAA*AAGCTT*GGAGGTTCCAGAAAATTGTACATGCAC-3' (sense)
		5'-AAA*GGTACC*TTAAGTTCCTGCATCACAAGAGCCAGT-3' (antisense)

References supplementary information:

(1) Mehta, A. Y.; Cummings, R. D. GlycoGlyph: a glycan visualizing, drawing and naming application. *Bioinformatics* **2020**, *36* (11), 3613-3614. DOI: 10.1093/bioinformatics/btaa190.

(2) Varki, A.; Cummings, R. D.; Aebi, M.; Packer, N. H.; Seeberger, P. H.; Esko, J. D.; Stanley, P.; Hart, G.; Darvill, A.; Kinoshita, T.; et al. Symbol Nomenclature for Graphical Representations of Glycans. *Glycobiology* **2015**, *25* (12), 1323-1324. DOI: 10.1093/glycob/cwv091.

(3) Baenziger, J. U.; Fiete, D. Structure of the complex oligosaccharides of fetuin. *Journal of Biological Chemistry* **1979**, *254* (3), 789-795.

(4) Inoue, S.; Inoue, Y. Chapter 7 - Fish glycoproteins. In *New Comprehensive Biochemistry*, Montreuil, J., Vliegenthart, J. F. G., Schachter, H. Eds.; Vol. 29; Elsevier, 1997; pp 143-161.

(5) Kim, J.; Ryu, C.; Ha, J.; Lee, J.; Kim, D.; Ji, M.; Park, C. S.; Lee, J.; Kim, D. K.; Kim, H. H. Structural and quantitative characterization of mucin-type O-glycans and the identification of O-glycosylation sites in bovine submaxillary mucin. *Biomolecules* **2020**, *10* (4), 636. DOI: 10.3390/biom10040636.

(6) Imre, T.; Kremmer, T.; Heberger, K.; Molnár-Szöllősi, É.; Ludanyi, K.; Pocsfalvi, G.; Malorni, A.; Drahos, L.; Vekey, K. Mass spectrometric and linear discriminant analysis of N-glycans of human serum alpha-1-acid glycoprotein in cancer patients and healthy individuals. *Journal of proteomics* **2008**, *71* (2), 186-197. DOI: 10.1016/j.jprot.2008.04.005.

(7) Ferragut, F.; Cagnoni, A. J.; Colombo, L. L.; Terrero, C. S.; Wolfenstein-Todel, C.; Troncoso, M. F.; Vanzulli, S. I.; Rabinovich, G. A.; Mariño, K. V.; Elola, M. T. Dual knockdown of Galectin-8 and its glycosylated ligand, the activated leukocyte cell adhesion molecule (ALCAM/CD166), synergistically delays in vivo breast cancer growth. *Biochimica et Biophysica Acta (BBA)-Molecular Cell Research* **2019**, *1866* (8), 1338-1352. DOI: 10.1016/j.bbamcr.2019.03.010.

(8) Kim, D. S.; Hahn, Y. The acquisition of novel N-glycosylation sites in conserved proteins during human evolution. *BMC bioinformatics* **2015**, *16* (1), 1-12. DOI: 10.1186/s12859-015-0468-5.

(9) Weide, T.; Herrmann, L.; Bockau, U.; Niebur, N.; Aldag, I.; Laroy, W.; Contreras, R.; Tiedtke, A.; Hartmann, M. W. Secretion of functional human enzymes by Tetrahymena thermophila. *BMC biotechnology* **2006**, *6* (1), 1-9. DOI: 10.1186/1472-6750-6-19.

(10) Rollenhagen, M.; Buettner, F. F.; Reismann, M.; Jirmo, A. C.; Grove, M.; Behrens, G. M.; Gerardy-Schahn, R.; Hanisch, F.-G.; Mühlenhoff, M. Polysialic acid on neuropilin-2 is exclusively synthesized by the polysialyltransferase ST8SiaIV and attached to mucin-type o-glycans located between the b2 and c domain. *Journal of Biological Chemistry* **2013**, *288* (32), 22880-22892. DOI: 10.1074/jbc.M113.463927.
